# Reproductive inequality in humans and other mammals

**DOI:** 10.1073/pnas.2220124120

**Published:** 2023-05-22

**Authors:** Cody T. Ross, Paul L. Hooper, Jennifer E. Smith, Adrian V. Jaeggi, Eric Alden Smith, Sergey Gavrilets, Fatema tuz Zohora, John Ziker, Dimitris Xygalatas, Emily E. Wroblewski, Brian Wood, Bruce Winterhalder, Kai P. Willführ, Aiyana K. Willard, Kara Walker, Christopher von Rueden, Eckart Voland, Claudia Valeggia, Bapu Vaitla, Samuel Urlacher, Mary Towner, Chun-Yi Sum, Lawrence S. Sugiyama, Karen B. Strier, Kathrine Starkweather, Daniel Major-Smith, Mary Shenk, Rebecca Sear, Edmond Seabright, Ryan Schacht, Brooke Scelza, Shane Scaggs, Jonathan Salerno, Caissa Revilla-Minaya, Daniel Redhead, Anne Pusey, Benjamin Grant Purzycki, Eleanor A. Power, Anne Pisor, Jenni Pettay, Susan Perry, Abigail E. Page, Luis Pacheco-Cobos, Kathryn Oths, Seung-Yun Oh, David Nolin, Daniel Nettle, Cristina Moya, Andrea Bamberg Migliano, Karl J. Mertens, Rita A. McNamara, Richard McElreath, Siobhan Mattison, Eric Massengill, Frank Marlowe, Felicia Madimenos, Shane Macfarlan, Virpi Lummaa, Roberto Lizarralde, Ruizhe Liu, Melissa A. Liebert, Sheina Lew-Levy, Paul Leslie, Joseph Lanning, Karen Kramer, Jeremy Koster, Hillard S. Kaplan, Bayarsaikhan Jamsranjav, A. Magdalena Hurtado, Kim Hill, Barry Hewlett, Samuli Helle, Thomas Headland, Janet Headland, Michael Gurven, Gianluca Grimalda, Russell Greaves, Christopher D. Golden, Irene Godoy, Mhairi Gibson, Claire El Mouden, Mark Dyble, Patricia Draper, Sean Downey, Angelina L. DeMarco, Helen Elizabeth Davis, Stefani Crabtree, Carmen Cortez, Heidi Colleran, Emma Cohen, Gregory Clark, Julia Clark, Mark A. Caudell, Chelsea E. Carminito, John Bunce, Adam Boyette, Samuel Bowles, Tami Blumenfield, Bret Beheim, Stephen Beckerman, Quentin Atkinson, Coren Apicella, Nurul Alam, Monique Borgerhoff Mulder

**Affiliations:** ^a^Santa Fe Institute, Santa Fe, NM 87501; ^b^Department of Human Behavior, Ecology and Culture, Max Planck Institute for Evolutionary Anthropology, Leipzig 04103, Germany; ^c^Department of Anthropology, University of New Mexico, Albuquerque, NM 87131; ^d^Department of Biology, University of Wisconsin, Eau Claire, WI 54702; ^e^Institut für Anthropologie und Anthropologisches Museum, University of Zürich, Zürich 8006, Switzerland; ^f^Department of Anthropology, University of Washington, Seattle, WA 98195; ^g^Departments of Ecology and Evolutionary Biology and Mathematics, University of Tennessee, Knoxville, TN 37996; ^h^International Centre for Diarrheal Disease Research, Dhaka 1000, Bangladesh; ^i^Department of Anthropology, Boise State University, Boise, ID 83725; ^j^Department of Anthropology, University of Connecticut, Storrs, CT 06269; ^k^Department of Structural Biology, Stanford University, Stanford, CA 94305; ^l^Department of Anthropology, University of California, Los Angeles, CA 90095; ^m^Department of Anthropology, University of California, Davis, CA 95616; ^n^Institute for Social Science, University of Oldenburg, Oldenburg 26129, Germany; ^o^Centre for Culture and Evolution, Brunel University, London UB8 3PH, United Kingdom; ^p^College of Veterinary Medicine, North Carolina State University, Raleigh, NC 27695; ^q^Jepson School of Leadership Studies, University of Richmond, Richmond, VA 23173; ^r^Institute for Philosophy, Justus-Liebig University, Giessen 35390, Germany; ^s^Department of Anthropology, Yale University, New Haven, CT 06520; ^t^Department of Global Health and Population, Harvard T.H. Chan School of Public Health, Boston, MA 02115; ^u^Department of Anthropology, Baylor University, Waco, TX 76706; ^v^Canadian Institute for Advanced Research, Toronto, CA M5G 1M1; ^w^Department of Integrative Biology, Oklahoma State University, Stillwater, OK 74078; ^x^College of General Studies, Boston University, Boston, MA 02215; ^y^Department of Anthropology, University of Oregon, Eugene, OR 97403; ^z^Department of Zoology, University of Wisconsin, Madison, WI 53706; ^aa^Department of Anthropology, University of Illinois, Chicago, IL 60607; ^bb^Department of Anthropology and Archaeology, University of Bristol, Bristol BS8 1QU, United Kingdom; ^cc^Department of Anthropology, Pennsylvania State University, University Park, PA 16802; ^dd^Department of Population Health, London School of Hygiene and Tropical Medicine, London WC1E 7HT, United Kingdom; ^ee^Department of Anthropology, East Carolina University, Greenville, NC 27858; ^ff^Department of Anthropology, Ohio State University, Columbus, OH 43210; ^gg^Department of Human Dimensions of Natural Resources, Colorado State University, Fort Collins, CO 80523; ^hh^Department of Evolutionary Anthropology, Duke University, Durham, NC 27708; ^ii^Department of the Study of Religion, Aarhus University, Aarhus 8000, Denmark; ^jj^Department of Methodology, London School of Economics and Political Science, London WC2A 2AE, United Kingdom; ^kk^Department of Anthropology, Washington State University, Pullman, WA 99164; ^ll^Department of Biology, University of Turku, Turku 20014, Finland; ^mm^Facultad de Ciencias Biológicas y Agropecuarias, Universidad Veracruzana, Veracruz 94294, Mexico; ^nn^Department of Anthropology, University of Alabama, Tuscaloosa, AL 35487; ^oo^Korea Insurance Research Institute, Seoul 150-606, Korea; ^pp^Department of Sociology, University of Massachusetts, Amherst, MA 01003; ^qq^Département d’Etudes Cognitives, Ecole Normale Supérieure, Université PSL, Paris 75230, France; ^rr^School of Psychology, Victoria University of Wellington, Wellington 6012, New Zealand; ^ss^Department of Biological Anthropology, University of Cambridge, Cambridge CB2 1TN, United Kingdom; ^tt^Department of Anthropology, Queens College (CUNY), New York, NY 11367; ^uu^Department of Anthropology, University of Utah, Salt Lake City, UT 84112; ^vv^Facultad de Ciencias Económicas y Sociales, Universidad Central de Venezuela, Caracas 1010A, Venezuela; ^ww^Department of Anthropology, Northern Arizona University, Flagstaff, AZ 86011; ^xx^Department of Psychology, Durham University, Durham DH1 3LE, United Kingdom; ^yy^Department of Anthropology, University of North Carolina, Chapel Hill, NC 27599; ^zz^SIT Graduate Institute, Brattleboro, VT 05301; ^aaa^Department of Biological Sciences, University of Cincinnati, Cincinnati, OH 45221; ^bbb^Economic Science Institute, Chapman University, Orange, CA 92866; ^ccc^National Museum of Mongolia, Ulaanbaatar 13373, Mongolia; ^ddd^School of Human Evolution and Social Change, Arizona State University, Tempe, AZ 85287; ^eee^SIL International, Dallas, TX 75236; ^fff^Department of Anthropology, University of California, Santa Barbara, CA 93106; ^ggg^Kiel Institute for the World Economy, Kiel 24105, Germany; ^hhh^Department of Animal Behaviour, Bielefeld University, Bielefeld 33615, Germany; ^iii^School of Anthropology and Museum Ethnography, University of Oxford, Oxford OX1 2JD, United Kingdom; ^jjj^Department of Anthropology, University College London, London WC1E 6BT, United Kingdom; ^kkk^School of Global Integrative Studies, University of Nebraska, Lincoln, NE 68588; ^lll^Department of Human Evolutionary Biology, Harvard University, Cambridge, MA 02138; ^mmm^Department of Environment and Society, Utah State University, Logan, UT 84322; ^nnn^Department of Economics, University of California, Davis, CA 95616; ^ooo^Nomad Science, Darkhad 67011, Mongolia; ^ppp^School of Ethnology and Sociology, Yunnan University, Yunnan 650106, China; ^qqq^School of Psychology, University of Auckland, Auckland 1010, New Zealand; ^rrr^Department of Psychology, University of Pennsylvania, Philadelphia, PA 19104

**Keywords:** reproductive skew, inequality, egalitarian syndrome, mating systems, monogamy

## Abstract

To address claims of human exceptionalism, we determine where humans fit within the greater mammalian distribution of reproductive inequality. We show that humans exhibit lower reproductive skew (i.e., inequality in the number of surviving offspring) among males and smaller sex differences in reproductive skew than most other mammals, while nevertheless falling within the mammalian range. Additionally, female reproductive skew is higher in polygynous human populations than in polygynous nonhumans mammals on average. This patterning of skew can be attributed in part to the prevalence of monogamy in humans compared to the predominance of polygyny in nonhuman mammals, to the limited degree of polygyny in the human societies that practice it, and to the importance of unequally held rival resources to women’s fitness. The muted reproductive inequality observed in humans appears to be linked to several unusual characteristics of our species—including high levels of cooperation among males, high dependence on unequally held rival resources, complementarities between maternal and paternal investment, as well as social and legal institutions that enforce monogamous norms.

## Significance

How and why do human systems of marriage and reproduction differ from comparable systems in other mammals? To answer these questions, we use data from 90 human societies and 49 mammalian species. We demonstrate that humans exhibit lower average sex differences in reproductive inequality than do most other mammals, while nevertheless falling within the mammalian range. We attribute these small sex differences in reproductive skew to institutions supporting monogamy, to a limited intensity of polygyny in those groups practicing it, and especially to heavy dependence of children on care from both parents in our species. Such mammal-wide comparisons reveal the extent of, and possible reasons for, human exceptionalism.

Debates over human exceptionalism are ubiquitous in the literature of the natural and social sciences (e.g., refs. 1–6). The extent to which individuals of the same sex in a given population differ in their fitness or reproductive success (e.g., number of surviving offspring) is commonly referred to as either reproductive skew or reproductive inequality (7), and human males have been frequently characterized as showing remarkably low levels of such skew. This equitable sharing of reproduction has been attributed variously to leveling norms (8, 9), coalitions (10), social interdependence (11), “gentlemen’s agreements” to reduce the costs of direct conflict (12, 13), intergroup dynamics (14–16), and gains to cooperative biparental investment (11) and/or male investment of rival material resources (e.g., territory or food that must be divided among offspring; refs. 17–21). While much has been made of this reproductive egalitarianism—the purportedly low level of reproductive skew in most human communities—few studies have actually estimated the extent to which both the absolute levels of reproductive inequality, and sex differences therein, differ between humans and the wider mammalian class. Even the simpler task of investigating variation in sex-specific reproductive inequality as a function of mating or the subsistence system in human populations has yet to be conducted in a systematic cross-cultural meta-analysis with individual-level data (but see ref. 22 for conceptually similar work).

Here, we aim to address this topic both theoretically and empirically. We begin by introducing a joint generative model of mating system and reproductive skew as a function of resource inequality and importance, which is based on a generalization of the polygyny threshold model (19, 20). We use the model to identify the parameter space where high reproductive skew is expected to emerge in each sex. The theoretical model grounds a suite of predictions about why humans might differ from nonhuman mammals in terms of reproductive inequality. We then introduce a large-scale comparative database of reproductive outcomes. The database contains individual-level reproductive records from 80,223 human individuals (male and female) from 90 extant and historical human societies—including foragers, horticulturalists, and pastoralists, as well as market-integrated rural communities—and comparable data from 49 species of (free-ranging) nonhuman mammals. We use a measure of skew—the multinomial index, *M*—that is not biased by mean reproductive success rate, age variation, or sample size (23), to robustly measure reproductive inequality in each dataset (*SI Appendix*, S1 for details). We then use Bayesian meta-analysis models to examine how reproductive skew in humans compares with that of other mammals generally—and nonhuman primates specifically. Following this, we also investigate how reproductive skew within humans varies as a function of subsistence mode. Both forms of comparative analysis help to evaluate outstanding explanations for apparent reproductive egalitarianism in humans. Additional phylogenetic models are presented in *SI Appendix*, S2.

## Theory and Hypotheses Regarding Reproductive Inequality.

Reproduction requires resources. Given the extent of, and variability in, material resource inequality across human societies (24), site-level metrics of reproductive skew might be expected to covary tightly with site-level metrics of material resource inequality. However, evolutionary social scientists have speculated that a low and relatively invariant degree of reproductive skew among men sets humans apart from most other mammal species and all other great apes (9, 10, 25–31). Proponents of this reproductive egalitarianism hypothesis assert that the need for within-group cooperation in humans mutes within-group reproductive competition among males (6, 14, 15, 32), even in social contexts where material resource inequality would be expected to lead to high levels of polygyny (20). A specific form of this claim is that reproductive egalitarianism is fundamentally linked to social norms that enforce monogamous pair-bonding in human societies, an idea that we refer to as the socially imposed monogamy hypothesis (14, 15). A further consideration that might account for human uniqueness lies in our sexual division of labor, in which men and women frequently provide complementary (i.e., nonsubstitutable) contributions to offspring, with males specifically acquiring and allocating rival resources (e.g., food, land, or time) that may be important for the rearing of offspring. This complementarity hypothesis posits that the fitness returns to male resource investment in offspring are a particularly important component of human trends toward monogamy, more limited levels of polygyny when it is present, and lower inequality in male reproductive success generally (11, 17–21).

**Fig. 1.**
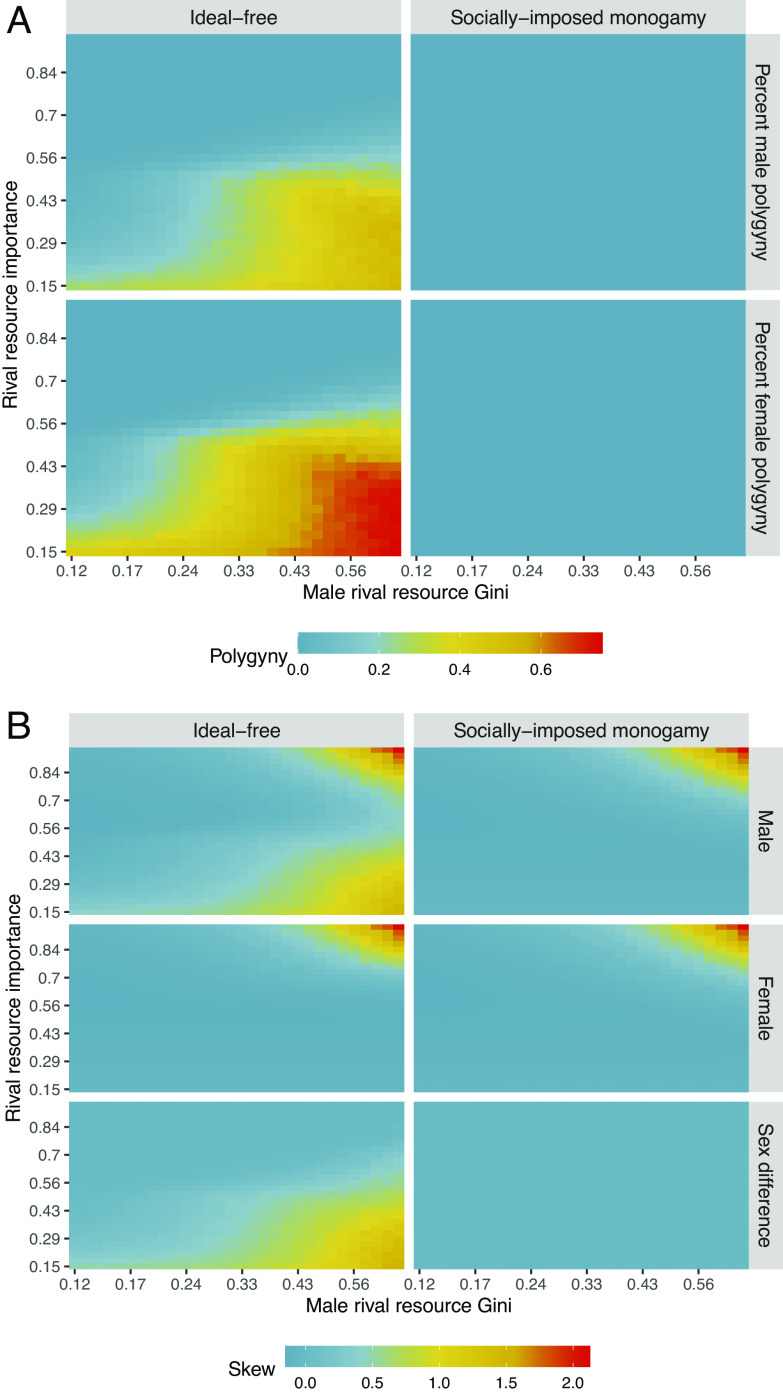
Polygyny intensity and reproductive skew as a function of male rival resource inequality, rival resource importance, and mating system norms using the generalized polygyny threshold model introduced by Oh et al. (19). Male rival resource inequality, *R*, is measured using the Gini coefficient and ranges ∈(0.12, 0.64). Rival resource importance, *μ*, is measured using the fitness elasticity of rival resources and ranges ∈(0.15, 0.95). Nonrival resources, *G*, are held constant with a Gini coefficient of 0.12. Nonrival resource importance, *γ*, is measured using the fitness elasticity of nonrival resources and is given by the equation *γ* = 1 − *μ* to ensure constant returns to scale. For further methodological and mathematical details, *SI Appendix*, S3. (*A*) Male polygyny (e.g., percentage of married men with more than one wife) and female polygyny (e.g., percentage of women with cowives) as a function of male rival resource inequality, rival resource importance to fitness, and mating system norms. (*B*) Reproductive skew, *M*, as a function of male rival resource inequality, rival resource importance to fitness, and mating system norms.

To address how changes in resource inequality and importance between species might explain the more equitable levels of skew in human societies, we draw on the mutual mate choice modeling framework introduced by Oh et al. (19). This generalization of the standard polygyny threshold model (33, 34) explores how the frequency of polygyny is expected to vary as a function of inequality in male and female resource holdings, the importance of these resource holdings to offspring recruitment, and the norms/constraints that govern the mating system (Fig. 1*A*). The model of Oh et al. (19) is based on a mating system governed by free female choice—each female chooses to pair with a willing male (who may or may not already be partnered) in such a way as to maximize her own fitness. This assumption—like most assumptions of this model—is not always met empirically (e.g., in humans, marriages can be arranged by parents against the fitness interests of their offspring). Nevertheless, the model is not designed to precisely describe reality but rather provide insight into how the differential importance of rival and nonrival resources impacts population-level reproductive inequality in a simple, hypothetical system.

Extending the Oh et al. (19) approach, we calculate how reproductive inequality is influenced by resource inequality, resource importance, and the mate-matching allocation given by the model (Fig. 1*B*). The model introduced by Oh et al. (19): i) accounts for the differential effects of rival resources (e.g., territory or food that must be divided among offspring) and nonrival resources (e.g., beneficial alleles that can be given to all offspring in equal measure) and ii) produces bilaterally Nash mate-matching outcomes—that is, it considers both male demand and female supply functions and ensures that no males and no females would prefer to change partners at equilibrium. The base model therefore yields an ideal-free distribution of females across males as a function of each male’s rival and nonrival resources and the importance (i.e., the fitness elasticity) of each type of resource to the recruitment of offspring. As is conventional (e.g., ref. 35), we present the model verbally as a system where males hold the rival resources critical to reproduction, and we assume that female inputs to offspring recruitment—like gestation, lactation, and time spent caring—are uniform across individuals. However, much of the model’s logic is extendable to systems where females are the holders of resources critical to reproduction, as we discuss later with regard to cooperative breeders.

As in the classic polygyny threshold model, it may be in the fitness interest of a given female to be the second mate of a sufficiently resource-rich male, rather than the singelton mate of a resource-poor male, for example. The assumption of free female choice in the Oh et al. (19) model, however, can be relaxed to investigate the effects that other methods of mate-matching would have on the frequency of polygyny and the extent of reproductive skew. Specifically, we consider a second model in which there is a social norm that restricts female choice, such that no female can choose to pair with a male who is already paired, leading to universal monogamy. For each model, we evaluate the level of skew in male and female reproductive success conditional on specified levels of rival and nonrival resource inequality and importance. The predictions of our models for polygyny and skew levels are provided in Fig. 1 *A* and *B*, respectively. We measure resource inequality using the Gini coefficient (36), a continuous measure of inequality between zero (each individual holds an equal share of a resource) and unity (a single individual holds all shares of a resource). We measure resource importance using fitness elasticity coefficients (37). For example, when the fitness elasticity of rival resources equals zero, fitness is invariant to rival resource holdings, but as the elasticity goes to unity, then fitness approaches proportionality to rival resource holdings. For further details on the definition of resource types (rival versus nonrival), the operationalization of resource importance to fitness, and the mathematical details of our model, *SI Appendix*, S3.

Fig. 1*A* shows how resource inequality and importance affect the extent of polygyny. Specifically, it demonstrates that when mating is ideal-free (*Left*-panels), the frequency of polygyny increases with rival resource inequality but decreases strongly with increasing rival resource importance. Socially imposed monogamy (*Right* panels) necessarily yields monogamy over the whole parameter space.

Fig. 1*B*—which should be read while referring to Fig. 1*A* for the degree of polygyny—depicts the consequences of resource inequality and importance for reproductive skew, revealing three key results. First, male skew is higher as rival resource inequality increases, but it is not coterminous with polygyny, insofar as it appears even in the monogamous parameter regimes. Male skew can either arise because i) polygyny increases variance in male mating success (see the *Lower-Right* region of the *Upper-Left* panel) or ii) because monogamy—either emergent or imposed—in contexts of high resource inequality and importance causes inequality in reproduction to approach proportionality to inequality in the distribution of rival resources (see *Upper-Right* regions in both *Upper* panels). Second, female skew is high only in contexts of monogamy, where important rival resources are unequally distributed (see *Upper-Right* regions of middle panels). Third, sex differences in skew emerge only in contexts where polygyny is prevalent (see the *Lower-Right* region of the *Lower-Left* panel).

In the following sections, we discuss the correspondence between the results of our model and known empirical patterns in nonhuman mammals broadly. We then formulate more detailed predictions about what separates human patterns of sex-specific reproductive inequality from those of nonhuman mammals. Specifically, we use the model to distinguish the predictions of the socially imposed monogamy hypothesis and the complementarity hypothesis.

## Model Results Are Consistent with Extant Nonhuman Data.

The results of our ideal-free model regarding the causes of high male reproductive skew are consistent with two well-known features of nonhuman mammal breeding systems.

First, reproductive variance among males who survive to breeding age is often associated with, and typically mediated by, variation in mate number (38–40). Variance in the reproductive success of males is generally higher than that of females, especially when there is polygyny (41, 42); however, this pattern does not always hold—e.g., when male rival resource inputs to offspring recruitment are important (43–46). These observations are consistent with our model, which shows that increased inequality in male rival resource holdings (e.g., where rival Gini ≳0.45) in the ideal-free condition leads to high frequencies of polygyny, as long as rival resource holdings are not essential inputs to offspring production (i.e., rival resource importance is low, *μ* ≲ 0.4). Polygyny, in turn, leads to higher male reproductive skew (due to higher variance in mate number) and lower female reproductive skew (due to more equal levels of rival resource provisioning per female) and thus to a positive sex difference in skew.

Second, exceptions to the general rule that male reproductive skew is higher under polygyny occur in cooperatively breeding species; in such species, variance in reproductive success is exceptionally high for both males and females, even though mating is generally monogamous (e.g., refs. 47–50). Among nonhuman mammals, cooperative breeding systems are often characterized by two properties: i) There is typically extremely high inequality in fitness-relevant resources—either social, material, or embodied [e.g., in dominance ranking, food access, or body size (see ref. 47, for a review)], and ii) there are steep fitness gradients to rival resource investment in offspring production. In fact, the energetic costs of breeding may frequently “be so high that a lone pair is effectively incapable of reproducing successfully” without the support of additional provisioners (51, p.91).

These two defining features of cooperative breeders directly map onto the parameters of our model. In the model, monogamy emerges when high rival resource inequality is present (e.g., rival Gini ≳0.45), and the importance of rival resources to offspring recruitment is also high (e.g., *μ* ≳ 0.7). Under this parameter regime, polygyny is eliminated because division of rival resources among multiple mates almost proportionately reduces the fitness of each of those mates; thus, mating investment costs disincentivize males from searching for additional partners (19). Reproductive skew, however, among both males and females increases nonetheless because inequality in offspring recruitment among the monogamous dyads approaches proportionality to the highly unequal distribution of rival resources—only those individuals with high resource levels can reproduce effectively. Where resources are both unequally distributed and critical to reproduction, monogamy can co-occur with high reproductive skew.

## Predictions for Skew in Humans and Nonhuman Mammals.

### Predicting overall differences in skew between humans and nonhuman mammals.

We start with the open question of whether reproductive skew in humans actually differs from that of nonhuman mammals more broadly. The literature on male reproductive skew provides a diverse set of expectations about differences in reproductive inequality between humans and nonhuman mammals. Predictions about differences in female reproductive skew, however, are comparatively rare.

One perspective views humans as yet another polygynous mammal, noting that over 80% of human societies allow polygynous marriage (e.g., ref. 17). While this observation is uncontroversial, and exceptionally large harems are seen in some populations or social classes (ref. 52), most men in most “polygynous societies” do not marry more than one woman (53, 54). Indeed, according to the Standard Cross-Cultural Sample, only 34% of societies are characterized by more than 20% of their men marrying more than one wife at a time (55, *SI Appendix*, S4). In foragers, for example, on average, only 14% of married men are polygynous, and only 21% of married women have cowives (56). Other sources of evidence, however, such as sexual dimorphism in body size (8, 57, 58), and Y chromosome evidence (59–61), do suggest a history of mild-to-moderate polygynous mating in our species (but see refs. 18 and 62). Stressing the continuity of polygynous mating systems between humans and other mammals [90 to 95% of whom exhibit some level of polygyny (63, 64)]—and overlooking, for the moment, our model-based results, which show that male skew does not necessarily arise only in polygynous contexts—the conventional prediction here is that levels of reproductive inequality among human males should fall within the range exhibited by other mammals.

The human reproductive egalitarianism hypothesis broadly, on the other hand, proposes that human males should stand out from most other mammals generally, and nonhuman primates specifically, in terms of their more equitable sharing of reproduction. This general hypothesis proposes that reproductive skew among human males, as well as sex difference in skew, will be lower than the same measures among most other mammals. The complementarity hypothesis goes farther and suggests that female reproductive skew in humans should actually exceed what is found in most other mammals, insomuch as the model suggests that female skew is increasing with the fitness importance of male rival resources and with the frequency of monogamy. In sum, we have predictions that

P1
  (a) male reproductive skew, *M*_*m*_, will be smaller,
  (b) female reproductive skew, *M*_*f*_, will be larger, and
  (c) sex differences in reproductive skew, *M*_*m*_ − *M*_*f*_, will be smaller in human populations than in nonhuman mammals generally, and nonhuman primates specifically.

### Predictions for nonhuman mammals based on mating system.

The majority of mammalian species exhibit some degree of polygyny (with only 5 to 10% exhibiting social monogamy, where mating is predominantly, though not exclusively, monogamous) (63, 64). Following the model, we expect monogamy in nonhuman mammals to arise primarily in contexts of high rival resource importance and inequality—as other mammals do not have normative or legal institutions to enforce monogamy. Thus, monogamy should—somewhat surprisingly—be associated with high absolute levels of skew for both sexes (43, 65). As such, we predict that

P2
  (a) male reproductive skew, *M*_*m*_, will be as large in monogamous species as in polygynous species,
  (b) female reproductive skew, *M*_*f*_, will be significantly larger in monogamous species than in polygynous species, and
  (c) sex differences in reproductive skew, *M*_*m*_ − *M*_*f*_, will be significantly smaller in monogamous species than in polygynous species.

### Predictions for human societies based on mating system.

In human societies, marriage, mating, and reproduction are distinct but related phenomena, in that socially recognized marriage practices structure but do not entirely dictate mating and reproduction (15, 66). However, given that most reproduction occurs within the context of recognized pair bonds [with generally less than 10% extra-pair paternity in most cases where it has been closely examined (67, 68), but ref. 69], marriage practices can provide an important—if indirect—window onto patterns of reproduction in human societies.

The socially imposed monogamy hypothesis suggests that norms and institutions promoting monogamous social unions are a key contributor to human reproductive egalitarianism (11, 12, 14, 15). Unlike in nonhuman mammals, monogamy in humans can be imposed, and so, monogamy in humans may arise under a broader range of resource inequality and importance regimes than in nonhuman mammals: That is, in humans, monogamy need not be associated with high rival resource importance. So, we predict

P3
  (a) Male reproductive skew, *M*_*m*_, will be lower in human populations with social institutions that mandate monogamy, than in human populations where polygyny is either socially tolerated or culturally normative.

To the extent that social imposition of monogamy constrains free female choice, thus increasing inequality in the extent of rival resource provisioning per wife, we expect that

P3
  (b) Female reproductive skew, *M*_*f*_, will be higher in human populations with social institutions that mandate monogamy, than in human populations where polygyny is either socially tolerated or culturally normative.

*SI Appendix*, S3.5 for quantitative estimates of the effect of socially imposed monogamy on sex-specific reproductive skew, holding constant resource importance and inequality levels.

As with nonhumans, monogamy—imposed or emergent—is expected to reduce sex differences in skew:

P3
  (c) Sex difference in reproductive skew, *M*_*m*_ − *M*_*f*_, will be lower in human populations with social institutions that mandate monogamy, than in human populations where polygyny is either socially tolerated or culturally normative.

At a finer scale, if the degree of sex difference in reproductive skew is causally linked to the number of marriages, then sex differences in skew should smoothly increase as the intensity of polygyny increases (at least when rival resource importance is not exceedingly high):

P3
  (d) Across human populations, there will be a continuous and positive relationship between sex differences in reproductive skew, *M*_*m*_ − *M*_*f*_, and the fraction of adult women who are polygynously married.

### Predictions from the complementary hypothesis.

One family of models developed to explain reduced levels of male reproductive inequality in humans emphasizes the role of complementary maternal and paternal investments in offspring (11, 17–21). These models suggest that insofar as reproduction in humans is constrained by fitness-relevant rival (i.e., zero-sum) and largely nonsubstitutable resources (e.g., labor, cattle, land, or time) provided by men to their wives and offspring, human polygyny will be muted in intensity and provide only limited fitness benefits to men, even when not formally prohibited by social institutions. Recognizing that males in most polygynous nonhuman mammals provide comparatively little in the way of rival resources to offspring, this complementarity hypothesis leads to the predictions that

P4
  (a) male reproductive skew, *M*_*m*_, will be lower,
  (b) female reproductive skew, *M*_*f*_, will be higher, and
  (c) sex differences in reproductive skew, *M*_*m*_ − *M*_*f*_, will be lower in polygynous human societies than in polygynous nonhuman mammals or polygynous nonhuman primates.

Each of these predictions supports the notion of complementarity in humans; first, marginal fitness returns to polygyny for males are expected to be low when offspring recruitment is limited by male resource investment, and so, male demand for polygyny will be low even when socially permitted, as long as paternal investment is important. Second, inequality in female fitness should be higher when male rival resources are unequally held and important to offspring recruitment, especially when social norms for more limited polygyny prevent women from distributing ideal-free and equalizing per capita rival resource access.

### Predictions from the socially imposed monogamy hypothesis.

The ideal-free model predicts high male skew, low female skew, and large sex differences in skew, for populations with high rival resource inequality (Gini ≳0.30) and low rival resource importance (*μ* ≲ 0.40). In contrast, the socially imposed monogamy model predicts that, even under such conditions, imposition of monogamy will lead to low male skew, low female skew, and small sex differences in skew. Although we have neither the Gini coefficients for resource measures nor the fitness elasticities needed to operationalize these predictions directly, we can use the subsistence system as a rough proxy for these measures until better data become available (24).

We assume that production systems based on land-limited agriculture are generally characterized by high levels of both rival resource inequality and importance (as found in the upper-right region of each frame in Fig. 1*A*) and that production systems based on foraging are generally characterized by low levels of both rival resource inequality and importance (as found in the lower-left region of each frame in Fig. 1*A*). As such, we expect high levels of monogamy and small sex difference skew in both subsistence modes.

Agropastoral systems, however, often feature high levels of resource inequality [with Gini coefficients ranging from 0.30 to 0.65 (ref. 20)] but lower fitness importance of rival resource per wife relative to what is observed in agricultural communities (70), creating opportunity for polygyny as seen in the *Lower-Right* region of the *Left*-most panels in Fig. 1*A*. Given this logic, the socially imposed monogamy hypothesis would predict that

P5 in human societies in which rival resource inequality is sufficiently high and rival resource importance sufficiently low (e.g., agropastoral societies), male and female reproductive skew, as well as sex differences in skew, will remain small in magnitude.

## Results

We test each of these predictions using a Bayesian meta-analysis with data from 90 human populations and 49 nonhuman mammals. To measure reproductive skew empirically, we use the multinomial index (23), *M*, a generalization of the opportunity for selection (41, 71), *I*, that adjusts for unequal exposure time to risk of reproduction (i.e., variation in the age at which reproductive success is measured). Interpretation of *M* is similar to that of Nonacs’ *B* (72, 73): *M* = 0 means that reproductive success is distributed as expected under a random multinomial model with equal reproductive success rates, *M* >  0 means that reproduction is positively skewed, and *M* <  0 means that reproduction is shared more equally than expected under a random multinomial model with equal reproductive rates.

### Prediction 1.

**Fig. 2.**
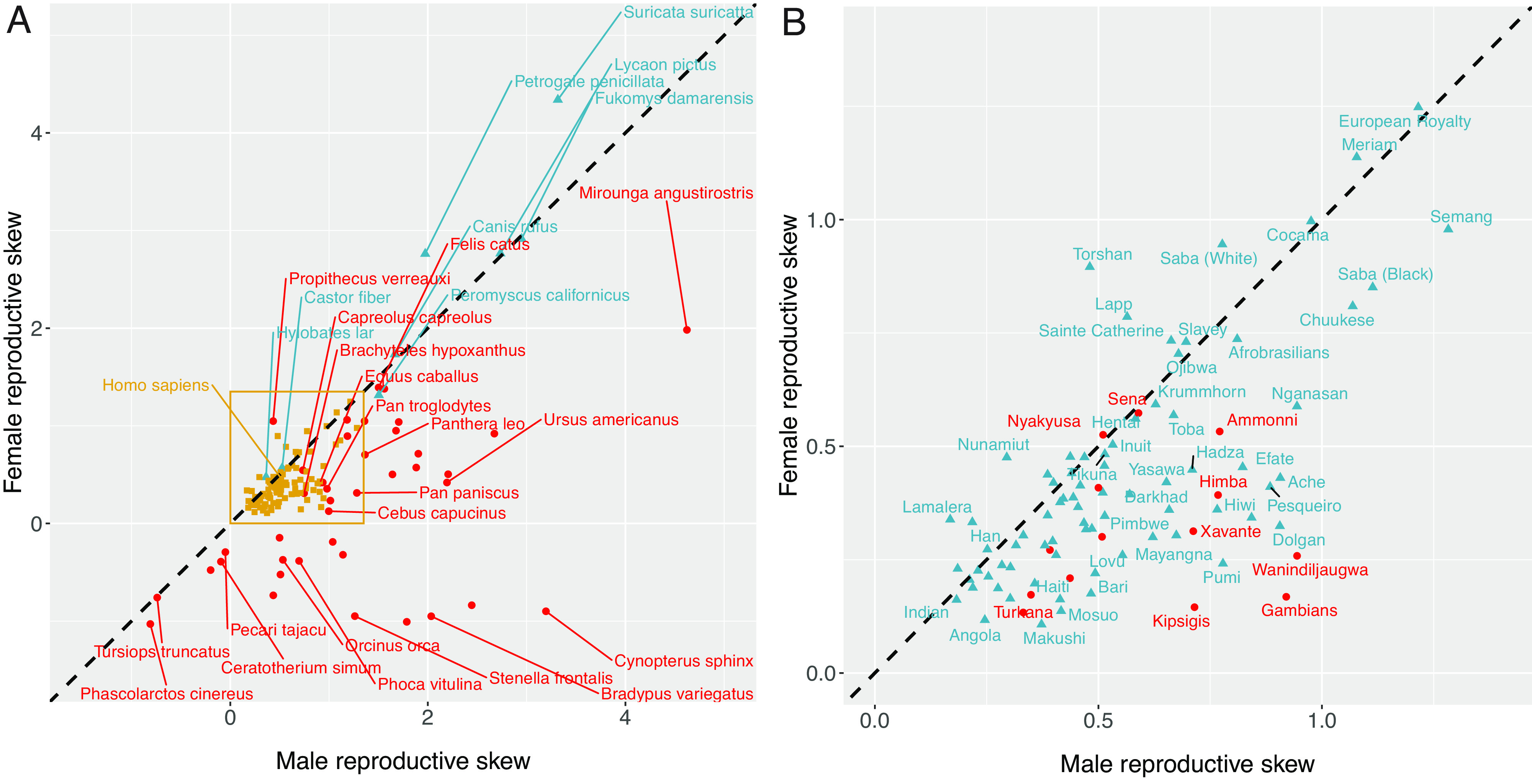
Raw data on male and female reproductive skew in mammals (*Left*) and humans (*Right*). In panel (*A*), polygynous nonhuman mammal values are plotted with red circles, monogamous nonhuman mammal values are plotted with blue triangles, and human values are plotted with goldenrod squares. In panel (*B*), polygynous human values are plotted with red circles, and monogamous human values are plotted with blue triangles. In both panels, points on the dashed diagonal line represent groups with equal male and female skew values. Points below the line indicate groups where male skew exceeds female skew, and vice versa for points above the line. Because *M* values are very high for some species, we visualize the data using the signed square root transform: $M^{*}=\;\text{sign}\left(M\right)\sqrt{\left|M\right|}$. (*A*) Male and female reproductive skew across species. (*B*) Male and female reproductive skew across human populations.

Fig. 2*A* visualizes the distribution of male and female reproductive skew values for all of the mammal species included in our dataset. Monogamous nonhuman mammals cluster in the upper right of the plot, where male and female reproductive skew values are similar, and reproduction is highly unequal. In contrast, polygynous nonhuman mammals show more variation, with some species showing high male skew and low female skew, and other species showing only slightly elevated male relative to female skew. In contrast to the wide distribution of skew values in nonhuman mammals, skew in human populations has a quite limited range. In particular, sex differences in reproductive skew are small among humans, and there is comparatively little variation across human populations, despite the fact that the populations in our sample differ markedly in terms of subsistence mode, mating system, and market integration. Fig. 2*B* presents a more detailed visualization of the human data. Polygynous human populations do tend to show higher male than female skew, but the size of the difference is limited in comparison to what is observed in mammals more broadly.

**Fig. 3.**
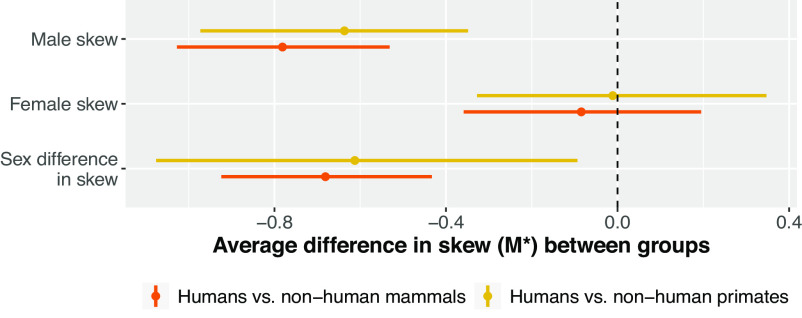
Posterior distributions of the difference in reproductive skew between humans and nonhuman mammals/nonhuman primates. Points represent posterior mean differences, and horizontal bars represent 89% credible regions. The dashed vertical line at zero indicates no difference. Humans stand out from both nonhuman mammals, generally, and nonhuman primates, specifically, in terms of having lower values of average male reproductive skew and lower sex differences in skew. Female reproductive skew, however, appears similar in humans and both nonhuman mammals and nonhuman primates—on average. Sample sizes: *N* = 90 human populations, *N* = 49 nonhuman mammal species, *N* = 12 nonhuman primate species.

As predicted in **P1(a)**, humans show reduced average levels of male reproductive skew, relative to both nonhuman mammals and nonhuman primates; Fig. 3. Regarding **P1(b)**, however, there is no evidence that human females show higher levels of reproductive skew when compared with either nonhuman mammals broadly, or nonhuman primates specifically. Finally, as predicted in **P1(c)**, humans do show significantly reduced average levels of sex differences in reproductive skew, relative to both nonhuman mammals and nonhuman primates.

Overall, Figs. 2 and 3 suggest that there is support for the reproductive egalitarianism hypothesis—specifically, with respect to male skew and sex differences in skew. However, human populations are by no means radical outliers in the mammalian class, clustering in a small range in the bivariate distribution of mammalian skew values—near average for female skew and moderately below average for male skew.

### Prediction 2.

**Fig. 4.**
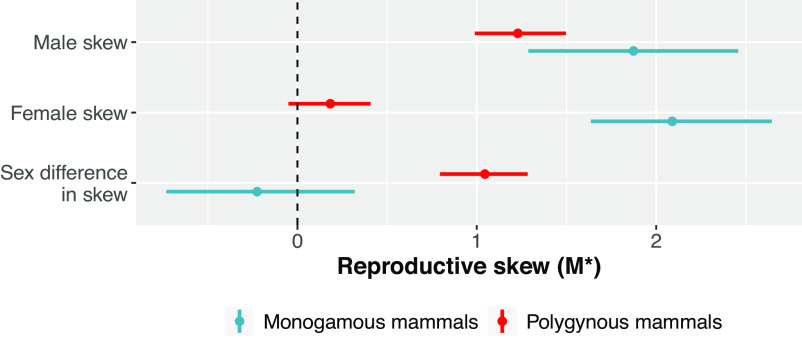
Posterior distributions of reproductive skew values (M^*^) in nonhuman mammals as a function of mating system. Points represent posterior means, and lines represent 89% credible regions. The dashed vertical line at M^*^ = 0 indicates that reproduction is neither positively skewed, nor more equal than would be expected under a random model. Monogamous nonhuman mammals stand out from polygynous nonhuman mammals, in terms of having significantly higher absolute values of male and female reproductive skew, and significantly lower sex differences in skew. Female reproductive skew, in particular, is strongly damped in polygynous species and elevated in monogamous species. Sample sizes: *N* = 49 nonhuman mammal species (8 monogamous, 41 polygynous).

As predicted in **P2(a)**, average male skew values appear to be as high, or even higher, in monogamous species as polygynous species; Fig. 4. Similarly, following **P2(b)**, average female skew values are dramatically higher in monogamous species than polygynous species. Finally, following **P2(c)**, there is no evidence of sex differences in skew among monogamous species, while there are substantial sex differences in polygynous species. These results are consistent with the idea that the presence of monogamy in nonhuman mammals is tightly linked to factors—like steep fitness gradients on resource provisioning—that increase reproductive skew in both males and females.

### Prediction 3.

**Fig. 5.**
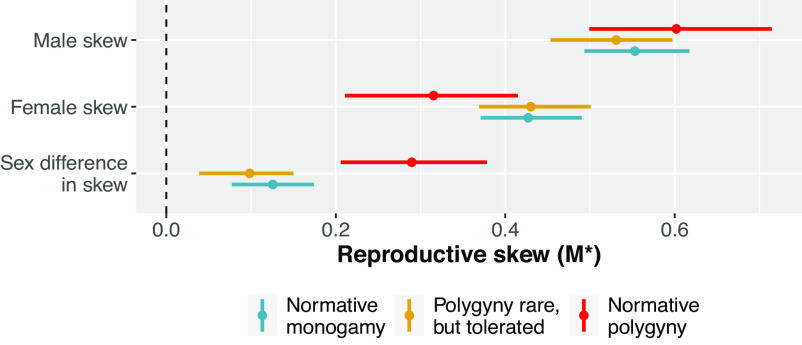
Posterior distributions of reproductive skew values (M^*^) in humans as a function of marriage system. Points represent posterior means, and lines represent 89% credible regions. The dashed vertical line at M^*^ = 0 indicates that reproduction is neither positively skewed, nor more equal than would be expected by a random model. In general, male reproductive skew appears fairly invariant to marriage system. Female skew appears slightly higher in human populations with socially imposed monogamy (normative monogamy) than populations in which polygyny is widely practiced (normative polygyny). Across all marriage system types, sex differences in skew are reliably different from zero—indicating that male reproduction is slightly more unequal than female reproduction, even where monogamy is imposed (normative monogamy) or frequent (polygyny rare, but tolerated). In contexts where polygyny is common, sex differences in skew are especially high. Sample sizes: *N* = 90 human populations (43 normative monogamy, 33 polygyny permitted, and 14 normative polygyny).

Insofar as only humans have institutional constraints on marriage practices, we test a set of hypotheses across our sample of human populations by contrasting groups that mandate monogamy and those that do not. Inconsistent with **P3(a)**, we find that male reproductive skew is not significantly lower in human populations with social institutions that mandate monogamy, compared to human populations where polygyny is socially tolerated or culturally normative (Fig. 5). Following **P3(b)**, there is some evidence that female skew is lower in contexts of normative polygyny than imposed monogamy (with a contrast of −0.11, 89%CI: −0.22, −0.02). Similarly, following **P3(c)**, there is evidence that sex differences in skew are higher in contexts of normative polygyny than imposed monogamy (with a contrast of 0.13, 89%CI: 0.05, 0.20).

**Fig. 6.**
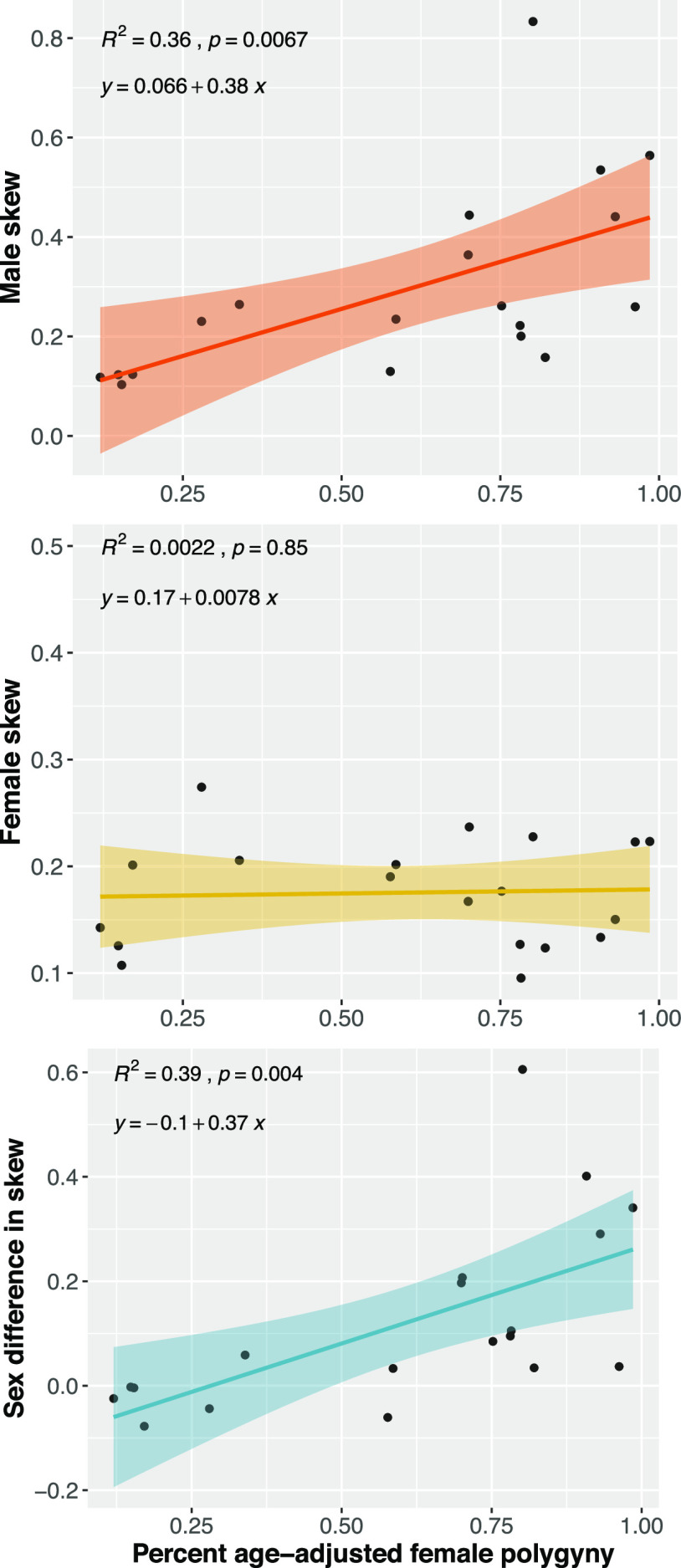
A strong positive relationship between male skew (*Top* frame) and sex differences in skew (*Bottom* frame), as a function of percent age-adjusted female polygyny in humans. Percent age-adjusted female polygyny is the predicted fraction of women married to men with more than one total wife by age 60 (see ref. 20, for details). The solid line plots the posterior mean regression, while the shaded area plots the 95% posterior credibility region. The black points give the data. Sample size: *N* = 19 human populations.

The marriage system data presented above, however, are based on rough site-level classifications and thus provide only a crude test of our predictions. To provide a more nuanced test, we draw on data from a subset of 19 human populations for which continuous measures of polygyny were available [from Ross et al. (20)] and linkable to male and female skew values. Fig. 6 plots the results. Consistent with **P3(d)**, we find that as the percentage of women with cowives increases, male reproductive inequality does tend to grow, as does the extent of sex differences in skew.

### Prediction 4.

**Fig. 7.**
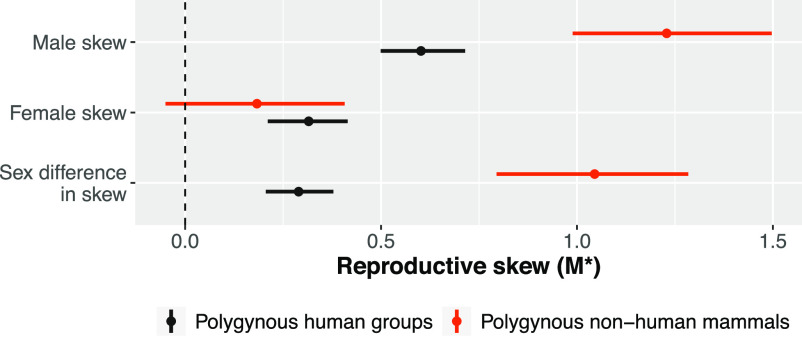
A different patterning of skew in polygynous human populations and polygynous nonhuman mammals. Points represent posterior means, and lines represent 89% credible regions. The dashed vertical line at M^*^ = 0 indicates that reproduction is neither positively skewed nor more equal than would be expected by a random model. Male reproductive skew in polygynous humans is substantially lower than in polygynous nonhuman mammals: The contrast is −0.59 (89%CI: −0.87, −0.32). Female skew is also higher in polygynous human populations than in polygynous nonhuman mammals: The contrast is 0.2 (89%CI: −0.05, 0.43). Sex differences in skew are therefore much lower in polygynous human populations than in polygynous nonhuman mammals: The contrast is −0.80 (89%CI: −1.03, −0.53). Sample sizes: *N* = 14 polygynous human populations and *N* = 41 polygynous nonhuman mammal species.

The complementarity hypothesis proposes that humans differ from most other mammals in the extent to which maternal and paternal care are jointly needed for offspring recruitment and that the need for such provisioning drives reduced reproductive skew in humans. To test this hypothesis, we compare skew in polygynous human populations to that in polygynous nonhuman mammals (Fig. 7). Consistent with **P4(a)**, we find that male reproductive skew is substantially lower in polygynous human populations compared to polygynous nonhuman mammals. Similarly, following **P4(b)**, female reproductive skew is noticeably higher in polygynous human populations compared to polygynous nonhuman mammals, though the size of the divergence is moderate, and the credible region slightly overlaps zero. Finally, following **P4(c)**, we find that sex differences in reproductive skew are substantially smaller in polygynous human populations compared to polygynous nonhuman mammals.

### Prediction 5.

**Fig. 8.**
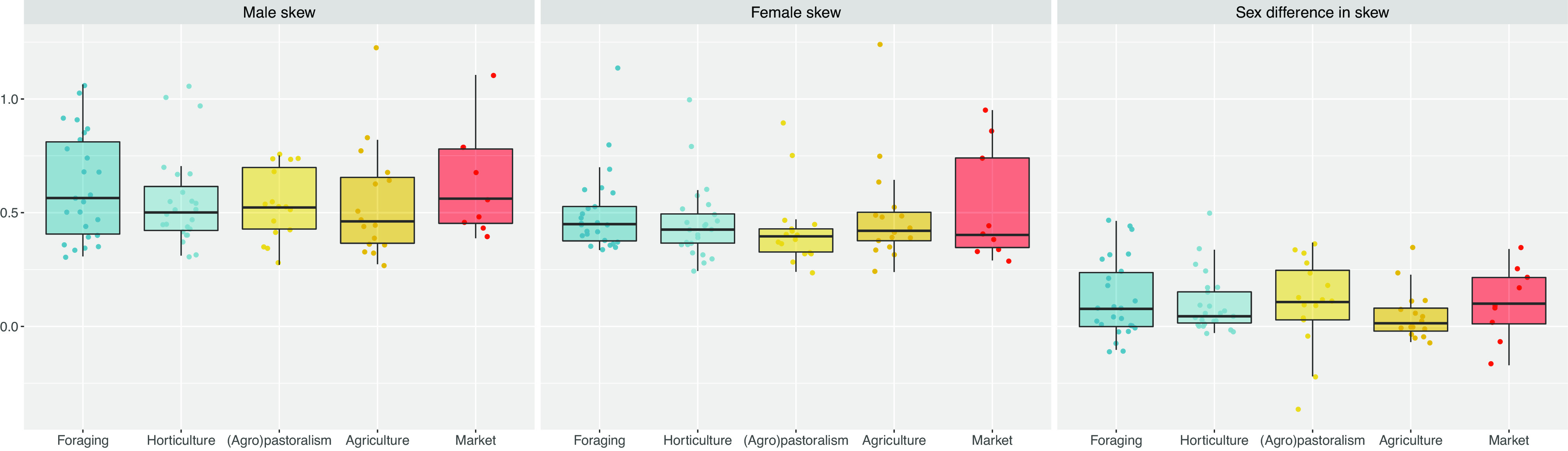
The distribution of sex-specific reproductive skew values as a function of subsistence mode in *N* = 90 human populations. We find that reproductive skew does not vary strongly by subsistence mode.

To examine the socially imposed monogamy hypothesis, we tested **P5**—if the intensity of reproductive skew varies predictably as a function of the subsistence system. Fig. 8 plots the results of this analysis. We find that male and female reproductive skew—as well as sex differences in reproductive skew—are more or less invariant to subsistence mode, paralleling some past work (22). We would not expect such a pattern to emerge if resource considerations (i.e., rival resource inequality and importance) alone determined mate-matching and reproductive output, suggesting a role for social norms that advocate monogamy (or at least more limited polygyny) in attenuating reproductive inequality across subsistence modes. Moreover, Table 1 shows that socially/normatively imposed monogamy is very common in agricultural and wage-based/market-integrated societies, which today account for a large share of the total human population.

**Table 1.** Marriage system as a function of subsistence system for the human populations in our dataset

	Normative monogamy	Polygyny rare, but tolerated	Normative polygyny
Foraging	10 (0.40)	13 (0.52)	2 (0.08)
Horticulture	7 (0.29)	14 (0.58)	3 (0.13)
(Agro)pastoralism	6 (0.37)	2 (0.13)	8 (0.50)
Agriculture	11 (0.69)	4 (0.25)	1 (0.06)
Market	9 (1.00)	0 (0.00)	0 (0.00)

Numbers represent the count of populations in each cross-tabulation; row-wise percentages are given in parentheses. Note that the distribution of subsistence and mating systems in our sample differs from that of the Standard Cross-Cultural Sample (*SI Appendix*, S4).

## Discussion

Here, we provide systematic evidence to support claims of extensive interspecies variability in reproductive skew using a metric, *M*, that is not biased by mean reproductive success rate, sample/group size, or differences in age structure (23). These analyses allow us to place human variation in reproductive inequality within the broader context of variation among mammals.

In line with the reproductive egalitarianism hypothesis, sex differences in reproductive skew in humans lie at the lower end of the mammalian and nonhuman primate distributions, a finding not apparent in a previous comparative study using only four human populations (74). This may be surprising, given that most human societies allow polygynous marriage and mating (75), and might therefore be expected to show high levels of reproductive inequality, particularly if males can retain multiple wives as they age (76). Some scholars have emphasized the extreme levels of reproductive inequality associated with despotism in humans (77), noting consistency with high reproductive skew in animal societies (78) and similar selection pressures on resource acquisition (79). Our analysis suggests, somewhat more subtly, that humans are a relatively unusual social mammal, in some ways more similar to mammals classed as socially monogamous rather than polygynous. This is consistent with von Rueden and Jaeggi’s (22) finding that the relationship between social status and reproductive success is significantly muted in small-scale human populations compared to many nonhuman primates. We discuss some of the methodological reasons why past research may have overemphasized the extent of reproductive inequality in our species in more depth in *SI Appendix*, S5.

Our analyses here extend past comparative work by unpacking the components of sex differences in skew and demonstrating the substantial role that variation in female reproductive success plays in driving sex differences in skew in both humans and nonhuman mammals. We also note that variation in reproductive success across human populations is much more limited than variation across species. There is, however, appreciable between-population variation in both male and female reproductive skew in humans (e.g., as a function of the marriage system), supporting many of Brown et al.’s (80) inferences, but now using a much more comprehensive database. Our data also suggest that between-population variation in reproductive skew is not well predicted by subsistence mode. While this finding appears to stand at odds with expectations derived from a mating market framework (19, 20, 33, 34), we find it quite plausible that there is substantial variation in social and material inequality within subsistence modes. Such variation may be shaped by institutional and economic particulars that are not well captured by a simple production-system typology (24, 81). To address this issue rigorously, we would require individual-level data on reproductive outcomes, marriage outcomes, and rival (e.g., money, land, or cattle) and nonrival (e.g., prestige) resource measures for both men and women, along with data on relevant cultural and institutional factors that might affect mating and reproduction.

At a broader scale, we show that sex differences in reproductive skew in nonhuman mammals are generally higher in mating systems characterized by polygyny, as opposed to monogamy, and that such sex differences in skew appear to be driven more strongly by the effects of polygyny on female reproductive variance than male reproductive variance. This pattern holds among humans too, though it is weaker in magnitude. By introducing a comparable reproductive skew index, *M*, and providing a set of comparable data, we open the door to future tests of a wide range of theoretical models aimed at explaining this within- and between-species variability in sex-specific reproductive skew (82).

### Human Reproductive Egalitarianism.

Sex differences in the strength of sexual selection (42, 74) are typically pronounced in large placental mammals because female reproductive rates are low, and significant parental tasks (e.g., gestation and lactation) can be provided only by females. These conditions create a strong opportunity for sexual selection, mediated through mating system (39). Because many human societies allow polygynous marriage and mating (75), the relative reproductive egalitarianism of humans is surprising, prompting the two hypotheses we introduced earlier—the complementarity hypothesis and the socially imposed monogamy hypothesis. We tested predictions derived from each hypothesis, and we found some support for both explanations.

Evidence for the complementarity hypothesis was somewhat mixed. We do find that male skew is smaller, and female skew is somewhat larger, in polygynous human populations compared to polygynous nonhuman mammals; however, we failed to find that human females overall show larger reproductive skew than nonhuman females. This latter finding arises because of the exceptionally high reproductive skew found in monogamous nonhuman mammals. If humans were characterized by high resource inequality and exceedingly high fitness gradients on rival resource provisioning (e.g., as is found in cooperative breeders), we would expect reproductive skew values in women to greatly exceed comparable values in most nonhuman mammals (i.e., with humans clustering closer to the monogamous nonhuman mammals in Fig. 2).

Favoring the complementary hypothesis, however, we find that even in human populations where polygyny remains culturally normative (and hence strict social imposition of monogamy is not occurring), sex differences in skew are considerably lower than in polygynous nonhuman mammals. Moreover, the positive reproductive skew observed among polygynous women suggests that resource provisioning per wife remains unequal, perhaps because the distribution of women across men deviates from that predicted by an ideal-free distribution. This finding is concordant with comparative data on polygyny and resource access cross-culturally, which indicate that men often marry many fewer wives than their material wealth would appear to allow (20, 83), presumably because the necessity of joint female and male provisioning of offspring reduces both male demand for, and female supply to, polygynous unions (19). It is also consistent with evidence of cowife rankings or favoritism (84).

A consequence of the idea that complementarity in male and female provisioning of important rival resources drives the evolution of monogamous mating/marriage systems (e.g., refs. 11, 17–21), however, is that rival resource elasticities high enough to generate monogamy simultaneously tend to predict high levels of reproductive skew in both sexes (e.g., as in cooperative breeding mammals). Humans generally show low reproductive skew and low mating/marriage skew (i.e., high levels of monogamy), which is a different pattern from what is found in cooperative breeding mammals. This seems to speak against the complementarity argument as a main/sole driver for human monogamy, but there are parameter ranges in our model—e.g., where rival resources are important, but not too unequally held—where monogamy emerges without inducing high absolute levels of skew for both sexes. Future work that assesses the model quantitatively with individual-level resource and reproduction data is thus needed to provide sharper tests of the complementarity hypothesis.

Regarding the socially imposed monogamy hypothesis, we find that reproductive skew levels in both sexes are surprisingly consistent across subsistence modes with substantially different levels of rival resource inequality and importance (20, 24, 70, 85). This finding is consistent with our previous suggestion that the ideal-free distribution assumptions of the polygyny threshold model are not being met—that is, there are some social constraints that limit polygyny even in contexts where rival resource inequality and importance might be expected to favor it. Clearly, socially imposed monogamy characterizes an increasing number of human populations, particularly those with agricultural or market-integrated economies. However, the specific prediction that male skew will be lower under normative monogamy relative to normative polygyny was not supported. Moreover, male and female skew values (and even sex differences in skew values) were essentially equivalent in societies where monogamy is social/legally imposed and in societies where polygyny is socially tolerated, but only rarely practiced. These findings—though somewhat surprising—are consistent with the model, which suggests that the absolute level of male skew can be substantial under both monogamous and polygynous parameter regimes, depending on resource inequality and importance levels, and the actual frequency of polygynyous unions. Even though many human societies allow polygyny, it is typical that within any given society, either few men actually marry polygynously or most men eventually marry multiple wives (e.g., ref. 86); neither scenario generates particularly high lifetime reproductive variance for men. Our more nuanced models—fit to the subset of data with a continuous measure of polygynous mating—do, however, suggest that male skew increases with the intensity of polygyny. Simplistic categorization of societies into mating system types can obscure this relationship. Nevertheless, sex differences in skew were indeed higher in societies with normative polygyny than in societies with either normative monogamy or infrequent but permitted polygyny.

In short, each of the two primary explanations for human reproductive egalitarianism offers only partial solutions to the paradox of reproductive skew decreasing, even as rival resource inequalities have grown substantially. Resource considerations alone appear insufficient to predict reproductive inequality, and there are likely a variety of cultural norms and institutional factors that constrain reproductive inequality, not just by prohibiting polygyny outright (as in socially imposed monogamy), but also in limiting the extent of polygyny even when permitted (20). Sexually transmitted infection burden (16, 87) and cowife conflict (88, 89), for example, have been proposed as factors that might select for more egalitarian marriage norms.

### Moving Forward.

Past work has raised the question of why monogamy and reproductive equality appear to be more common in human populations than nonhuman mammals. A leading explanation has been that norms for monogamy—or at least less intensive polygyny—reduce the frequency and intensity of male–male competition within groups (12, 13) and spread as a result of intergroup competition (14, 15) or some form of cultural group selection (90); the data presented here are somewhat consistent with such arguments, insomuch as monogamy is the prevailing marriage form in agricultural and market economies, which today are demographically predominate. However, our finding that male reproductive skew does not appear to strongly covary with the mating system in humans suggests that we must look beyond the interests of males when attempting to explain the patterning of reproductive skew in our species. Although there are smaller sex differences in reproductive skew in the monogamous populations in our sample relative to the normatively polygynous ones, this pattern appears to be driven as strongly by elevated skew among monogamous women as by reduced skew among monogamous men.

Like men, women can benefit reproductively from serial mating and marriage by securing the support of multiple provisioners (46, 91–93), especially in contexts of partible paternity beliefs (e.g., refs. 94 and 95). Under conditions of sharp rival resource inequalities, monogamy can promote intense competition among women, for example, through payments of dowry (96), thereby potentially increasing female reproductive skew. Examining competition and cooperation within each sex (e.g., ref. 97) may ultimately shed more light on the patterning of reproductive skew and provide a fuller explanation for why the sex differences in skew measured here are so sensitive to variation in female reproductive success.

Additionally, the reproductive benefit of polygyny to males can be reduced under conditions of promiscuity or polygynandry (39, 98, 99) or where females reproduce very frequently (100). For example, Bergeron et al. (101) show that polygynous cercopithecine primates have relatively low sex differences in skew in the context of promiscuity and polygynandry, and Lukas and Clutton-Brock (100) find lower male reproductive skew in species where females breed frequently. In addition, marriage systems do not entirely dictate mating and reproduction among humans (15, 66). For example, Prall and Scelza (99) show that, despite the fact that the Himba are nominally polygynous, many women openly maintain nonmarital sexual partnerships. Such practices may mute reproductive inequality among men, even in societies that are purportedly polygynous (99).

The evolutionary origins of social monogamy in many nonhuman mammals are thought to lie in female scarcity and low densities (102, 103); however, resource considerations generally—and complementarities specifically—also appear to be an important factor promoting cooperative pair-bonds, both in cooperatively breeding mammals (e.g., ref. 47) and in birds and fishes (104, 105). Among mammals, groups such as the callitrichids take advantage of returns to scale in investment from females and males (106). Similarly among humans, and unlike most nonhuman primates, strong complementarities in parental care, together with high fitness returns to the provision of care by parents and alternative caretakers, may be key to both the relatively minor sex differences in reproductive skew in our species and the variability in skew across human populations. Our model demonstrates the potential importance of complementarity in generating monogamy, but both the model and data presented here show that monogamy does not necessarily result in low skew for either sex. As such, future empirical work, integrating individual-level data on reproduction, marriage, and resource holdings is needed to provide finer-scale tests of the model.

### Conclusions.

The multinomial index, *M*, provides a metric for directly comparing skew across datasets with substantial differences in mean reproductive success, group/sample size, and age structure (23). The analytic relationship between *M* and the binomial index, opportunity for selection, coefficient of variation, standard deviation, and variance allowed us to compile comparable data from a large number of published skew values in nonhuman mammals.

Using this index, we analyzed a carefully constructed dataset to test whether reproductive inequality in a large sample of human societies differs from that observed in other mammals, generally, and nonhuman primates, specifically. We find that humans exhibit lower skew among males, and smaller sex differences in skew, than most other mammals, while nevertheless falling within the mammalian range. These low values can be attributed in part to the prevalence of monogamous marriage in humans, compared to the predominance of polygyny in nonhuman mammals, and in part to the limited intensity of polygyny where it is practiced.

## Materials and Methods

### Data Inclusion.

We compiled data on reproductive outcomes in human populations by reviewing the published literature on variation in human reproductive success and inviting anthropologists who had published on the topic to submit individual-level data (derived from a random sampling or census methodology) for this analysis. Additional data were gleaned from genealogical datasets published by KinSources (107). *SI Appendix*, S6, for a complete list of populations, sources, and citations. *SI Appendix*, S10, for details on research permissions, ethics reviews, and informed consent procedures.

To classify the marriage system of each population, we used standardized categories: normative monogamy, socially tolerated (but typically rare) polygyny, or culturally normative polygyny (e.g., when > 20% married women are married polygynously). We relied on ethnographers’ qualitative measures/judgments or ethnographic descriptions in the primary literature, to code the data. Subsistence mode was also provided by ethnographers or coded on the basis of published literature. Both marriage system and subsistence system codes are reductive categories, and there is substantial variation within classes; future research would benefit from individual-level data on marriage practices and subsistence time allocation. Similarly, reproductive success data are typically collected via self-reports, and so, extra-pair paternity could distort estimates of skew; future work may wish to compare self-reports of reproductive success with true paternity data (see ref. 69). To test whether our results are robust to dropping populations with small samples, or less rigorous demographic protocols, we replicate our analysis, including only 29 populations—the subset for which sample size was large and the data were collected for the purposes of demographic analyses. *SI Appendix*, S8, which show that our qualitative findings hold.

We compiled data on nonhuman mammal populations by performing a systematic search in Google Scholar and reviewing papers that included the term “reproductive skew.” Additionally, we reviewed all empirical articles on mammals that cite Nonacs (73). We also located data cited by Lukas and Clutton-Brock (100) and referred to the original data cited therein. Finally, we invited field researchers to contribute unpublished individual-level data on reproductive outcomes. *SI Appendix*, S6, for sources and citations. We attempted to restrict our nonhuman mammal dataset to high-quality estimates (e.g., samples for which paired molecular data on male and female reproduction were available). Data based simply on observed rates of copulation were deemed of limited value for assessing reproductive skew and therefore never integrated into the dataset or analyses.

### Statistical Modeling.

Methodological and mathematical details are provided in *SI Appendix*, S7. Model code and data are provided as supplementary files. The full workflow from raw data (cleaned of missing cases and stripped of identifying information) to estimation of *M*, meta-analysis, model diagnostics, and visualization of results is available to anyone wishing to reproduce or extend the analyses: https://github.com/ctross/reproductiveskew. Because *M* values are very high for some species, we visualize and analyze the data using the signed square root transform: $M^{*}=\mathrm{sign}\left(M\right)\sqrt{\left|M\right|}$, which yields more normally distributed data.

All data processing is handled using the R software environment (108). All statistical models are coded in the Stan (109) language and are fit using Hamiltonian Monte Carlo (110). The Stan C++ Library is accessed using the rstan package.

## Supplementary Material

Appendix 01 (PDF)

## Data Availability

All study data are included in the article and/or supporting information.
