# Supplementary material for: Reproductive inequality in humans and other mammals
Source: Proc Natl Acad Sci U S A. 2023 May 22;120(22):e2220124120. doi: 10.1073/pnas.2220124120 (PMC10235947; doi:10.1073/pnas.2220124120)
Supplement: Supplementary file 1 — Appendix 01 (PDF) [file pnas.2220124120.sapp.pdf]

Supplementary Materials:
Reproductive inequality in humans and other mammals

Cody T. Ross, Paul L. Hooper, Jennifer E. Smith, Adrian V. Jaeggi, Eric Alden Smith, Sergey Gavrilets, Fatema tuz Zohora, John Ziker, Dimitris Xygalatas, Emily E. Wroblewski, Brian Wood, Bruce Winterhalder, Kai P. Willführ, Aiyana K. Willard, Kara Walker, Christopher von Rueden, Eckart Voland, Claudia Valeggia, Bapu Vaitla, Samuel Urlacher, Mary Towner, Chun-Yi Sum, Lawrence S. Sugiyama, Karen B. Strier, Kathrine Starkweather, Daniel Major-Smith, Mary Shenk, Rebecca Sear, Edmond Seabright, Ryan Schacht, Brooke Scelza, Shane Scaggs, Jonathan Salerno, Caissa Revilla-Minaya, Daniel Redhead, Anne Pusey, Benjamin Purzycki, Eleanor A. Power, Anne Pisor, Jenni Pettay, Susan Perry, Abigail E. Page, Luis Pacheco-Cobos, Kathryn Oths, Seung-Yun Oh, David Nolin, Daniel Nettle, Cristina Moya, Andrea Bamberg Migliano, Karl J. Mertens, Rita A. McNamara, Richard McElreath, Siobhan Mattison, Eric Massengill, Frank Marlowe, Felicia Madimenos, Shane Macfarlan, Virpi Lummaa, Roberto Lizarralde, Ruizhe Liu, Melissa A. Liebert, Sheina Lew-Levy, Paul Leslie, Joseph Lanning, Karen Kramer, Jeremy Koster, Hillard S. Kaplan, Bayarsaikhan Jamsranjav, A. Magdalena Hurtado, Kim Hill, Barry Hewlett, Samuli Helle, Thomas Headland, Janet Headland, Michael Gurven, Gianluca Grimalda, Russell Greaves, Christopher D. Golden, Irene Godoy, Mhairi Gibson, Claire El Mouden, Mark Dyble, Patricia Draper, Sean Downey, Angelina L. DeMarco, Helen Elizabeth Davis, Stefani Crabtree, Carmen Cortez, Heidi Colleran, Emma Cohen, Gregory Clark, Julia Clark, Mark A. Caudell, Chelsea E. Carminito, John Bunce, Adam Boyette, Samuel Bowles, Tami Blumenfield, Bret Beheim, Stephen Beckerman, Quentin Atkinson, Coren Apicella, Nurul Alam, Monique Borgerhoff Mulder

1 The Multinomial Index 2
- 1.1 Definition . . . 2
- 1.2 What M measures . . . 2
- 1.3 Software implementation . . . 3

2 Phylogenetic analyses 3

3 Modeling reproductive skew as a function of resource inequality and importance 4
- 3.1 Resource types . . . 5
- 3.2 Resource inequality . . . 6
- 3.3 Resource importance to fitness . . . 6
- 3.4 Simulating marriage market outcomes and reproductive skew . . . 7
- 3.5 Effects of socially imposed monogamy versus ideal-free mate matching on reproductive skew . . . 8

4 Polygyny in the Standard Cross-Cultural Sample 8

5 Reproductive egalitarianism in humans 8

6 Data Sources 9

7 Meta-analysis of reproductive skew in mammals 16
- 7.1 Model definition . . . 16
- 7.2 Software implementation . . . 16
- 7.3 Model fit: Estimation of M . . . 17
- 7.4 Model fit: Comparative models . . . 17

8 Robustness check 17

9 Extended Acknowledgments 23

10 Statements regarding research approvals/permissions 24
- 10.1 Data from published sources . . . 24
- 10.2 Data from co-authoring researchers . . . 26

1 The Multinomial Index

1.1 Definition

Assuming we have data on an RS (reproductive success) measure, r, and some age or "exposure time" measure, t, from a sample of N individuals, then $M(r,t)$ is defined as:

$$M(r,t) = \check{M}(r,t) - \mathbb{E}[\check{M}(X,t)] \tag{1}$$

where:

$$\check{M}(r,t) = \frac{N}{O^2} \sum_{i=1}^{N} (r_i - \bar{r}_i)^2 \tag{2}$$

and where:

$$X \sim \text{Multinomial}\left(O, \frac{t}{T}\right) \tag{3}$$

Eq. 1 defines $M(r,t)$ to be the difference of the observed estimate of $\check{M}(r,t)$ from its expected value if RS were distributed as a multinomial outcome with the same sample size, average RS rate, and exposure time vector. Eq. 2 then defines $\check{M}(r,t)$, an extension of the opportunity for selection [1, 2], I, that adjusts for unequal exposure time to risk of RS. O is the total number of offspring produced by a sample of N individuals, T is the total exposure time contributed by all N individuals, r_i is the number of offspring produced by individual i, and $\bar{r}_i = \frac{R}{T} t_i$ is the expected number of offspring that individual i would have produced at his or her age if reproductive rates were perfectly equal within the group. Interpretation of M is similar to that of B: $M = 0$ means that RS is distributed as expected under a random multinomial model with equal RS rates, $M > 0$ means that reproduction is positively skewed, and $M < 0$ means that reproduction is shared more equally than expected under a random multinomial model with equal RS rates.

The multinomial index, M, is analytically related to Nonacs' binomial index, B [3, 4], the coefficient of variation, and the opportunity for selection, I [1], and conceptually related to the Gini coefficient [5]. Like Nonacs' B, it adjusts for variation in the amount of time in which individuals have been exposed to risk of RS. Advantageously, it is not affected by differences in sample size that have hindered past efforts to compare skew across study populations. Specifically, M does not show the strong inverse relationships that B, λ, and "the proportion of total reproduction achieved by the most successful individual" show with group/sample size. The significant negative relationship between the number of males in a group and skew in male mating success reported among primates [6], for example, disappears when employing M [see re-analysis in: 7]. M also corrects a negative dependence of both I and the coefficient of variation on mean reproductive success.

See Ross et al. [7] for derivation, mathematical details, simulation tests, and an empirical test of mating skew using M.

1.2 What M measures

The analytical relationships between M, B, I, the coefficient of variation, the standard deviation, and the variance have allowed us to draw on a large literature of published skew values in

non-human mammals, and compare them using a standard metric.

M can be used to measure skew in the rate of mating (by passing in a vector of mating event data), skew in the rate of production of offspring (by passing in a vector of fertility data), or skew in the rate of offspring recruitment (by passing in a vector of surviving offspring data). A more detailed discussion is provided in Ross et al. [7]. There, we state that: "...care should be taken regarding both sampling frame and function inputs. RS data can be defined as offspring ever produced—reflecting inequality in fertility—or as offspring recruited to reproductive age—reflecting inequality in both fertility and recruitment. Data on age/exposure time may be passed into M—so that M reflects inequality in reproductive rate while living—or age/exposure time may be held fixed across individuals—so that M reflects inequality in lifetime RS. If a complete census of individuals is not constructed, estimates of skew might be impacted by sampling design and/or dropout due to differential mortality. In short, estimates of M will reflect different quantities based on the choice of input variables, sampling design, and other data inclusion criteria, as is necessarily true of any existing or potential skew measure."

For the ethnographic samples included with this publication, M yields a reproductive skew measure that reflects *heterogeneity in rates of reproduction among currently living adults*. In the historical data included here, M yields a reproductive skew measure that reflects *heterogeneity in rates of reproduction among all adults*. In the non-human populations included here, some estimates were based on lifetime reproductive success measures (LRS; e.g.[8]), and other estimates were based on heterogeneity in rates of reproduction among living adults over a fixed time window. There is some variation across samples in what M is measuring, but such measurement error is essentially unavoidable in a comparative project this broad.

1.3 Software implementation

The `SkewCalc` R package provides a means of calculating the multinomial index from data on exposure time and reproductive success across individuals within a sample. This package uses Bayesian methods to estimate confidence intervals for $\check{M}$ and M. These methods are described in Ross et al. [7].

The `SkewCalc` R package can be downloaded, or installed directly, from: `https://github.com/Ctross/SkewCalc`. See the readme file on GitHub to install.

2 Phylogenetic analyses

To conduct initial species-level phylogenetic analyses, we downloaded the mammalian phylogeny published by Upham et al. [9], and pruned the tree to include only the species included in our database. Fig. 1 plots the phylogeny of species included in our database, and presents the outcome and predictor variables using a heatmap. We then test for phylogenetic signal in male skew, female skew, and sex differences in skew using both Pagel's λ [10] and Blomberg's K [11], via the `phytools` R package [12]. Table 1 provides the results.

Polly [13] argues that λ can be thought of as a scaling factor so that a phylogenetic tree fits a Brownian motion model. Values of Pagel's λ near 1, indicate that the structure of the phylogeny can explain the outcome distribution, assuming a simple Brownian motion model of

trait evolution. Values of Pagel's λ near 0, on the other hand, indicate that the phylogeny has to become a 'star phylogeny' in order to explain the data under a Brownian motion model. We find intermediate, but non-significant values of $\lambda \approx 0.46$ for male and female reproductive skew, and a value of $\lambda \approx 0$ for sex differences in reproductive skew, indicating little robust evidence of phylogenetic signal.

Similarly, Polly [13] argues that K can be thought of as the proportion of the covariance that is due to phylogeny. Values of Blomberg's [11] $K < 1$ indicate that species resemble each other—with respect to some outcome variable—less than would be expected under a Brownian motion model of trait evolution. Greater values of K are thus indicative of stronger phylogenetic signal. In our data, we find values of $K \approx 0$, indicating little evidence of robust phylogenetic signal.

Because these initial models suggest little robust evidence of phylogentic signal in species-level outcomes, and because other methodological issues—like the incomplete sampling of mammalian species—limit our analyses, we focus on simple descriptive comparisons between estimates of reproductive skew in human populations and non-human mammal species. In future work, other researchers may wish to apply more nuanced phylogentic models to these data.

Similarly, although some new methods to deal with phylogenetic relationships between human cultural groups have been developed [14] and deployed [15], and new cultural supertrees [16] have been released, the application of phylogenetic methods to behavioral variation in human populations remains a hotly debated topic [17]. As such, we present our analyses here in descriptive terms, and leave cultural phylogenetic analyses for future work.

Table 1: *Tests for phylogenetic signal using Pagel's λ [10] and Blomberg's K [11]. There is no evidence of significant phylogenetic signal using either metric.*

Sex	Metric	Value	P
Male M	λ	0.47	0.29
Female M	λ	0.45	0.61
Sex difference	λ	0.00	1.00
Male M	K	0.12	0.11
Female M	K	0.08	0.49
Sex difference	K	0.05	0.88

3 Modeling reproductive skew as a function of resource inequality and importance

Our model of reproductive skew is based very closely on the model of mutual mate choice introduced in Oh et al. [18]. We present a review of the model here for completeness, but refer interested readers to the original publication for full details, justifications, and derivations.

Figure 1: *Phylogenetic tree of the species included in our database. Traits are represented on the tips of the tree using a heatmap. Monogamy is a binary variable (blue represents monogamy, and red represents some form of polygyny). Each M value (Male, Female, and Difference) is a continuous measure of reproductive skew (or sex difference in reproductive skew); see legend.*

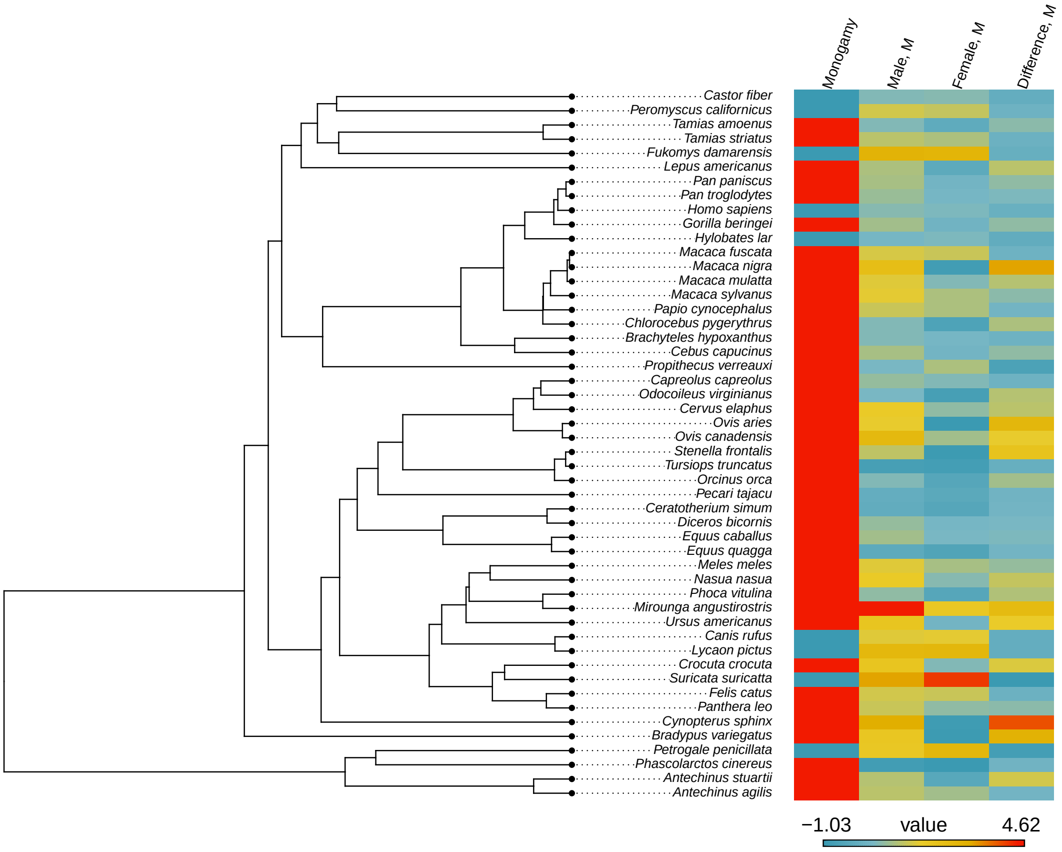

3.1 Resource types

We consider fitness-relevant resources, l, held by each female, and two types of fitness-relevant resources held by each male: non-rival resources, denoted as g_i, and rival resources, denoted as m_i. Male resources are variable across individuals, so we index them by i. A resource is rival if its consumption by one individual prevents consumption by another: e.g., food eaten by one individual cannot be eaten by another. In contrast, a resource is non-rival if its consumption by one individual does not prevent consumption by another: e.g., traditional ecological knowledge can be passed on from a human parent to multiple children at the same time. Rival resources—such as land, livestock, territory, or food—must be divided among the offspring of a polygynous father. Non-rival resources, in contrast, are analogous to a public good, in that the amount

inherited by one's offspring is independent of their number; a male's alleles for a preferred or adaptive phenotype are an example of a non-rival resource in the context of offspring production, as all offspring of a given male can inherit them.

If mating costs are null, then the male non-rival and rival resources available to any particular partner are g_i and $\frac{m_i}{n_i}$, respectively, where n_i is the number of partners of individual i. If mating costs are not null, then we can represent the total mating investment devoted to acquiring a partner by a cost equal to c units of the rival resource per partner. The remaining rival resources available to each partner can be written as: $\frac{m_i - cn_i}{n_i}$. In general, we assume that rival resources are allocated equally among partners who are themselves identical, and who in turn do not differentiate investment among their offspring. Our model below aims to disentangle the effects of variability in male rival and non-rival resources on reproductive skew, holding constant female resources at a fixed level, l.

3.2 Resource inequality

We measure resource inequality using the Gini coefficient [19], a continuous measure of inequality between zero (all individuals hold equal shares of a resource) and unity (a single individual holds all shares of a resource).

3.3 Resource importance to fitness

We assume that the fitness of each male is a function of his own rival and non-rival resource inputs, female resource inputs, and the number of partners he matches with. Fitness is generated according to a Cobb-Douglas production function [20]. A male's fitness, denoted by w_i, can be described as the number of partners times the fitness of each:

$$w_i = \underbrace{n_i}_{\text{Partners}} \cdot \underbrace{l^{\lambda} g_i^{\gamma} \left(\frac{m_i - n_i c}{n_i} \right)^{\mu}}_{\substack{\text{Fitness} \\ \text{per partner}}} \tag{4}$$

In this model of offspring production, the terms λ, γ, and μ determine the importance of female resource holdings, and male non-rival and rival resource holdings in determining fitness. These parameters are *elasticities*; that is, they measures the percentage change of one variable (i.e., fitness) in response to a percentage change in another (e.g., rival resources). Here, we assume that the resources contributed to reproduction by each female is uniformly l, and so females do not vary in terms of resource holdings.[1] Because females are assumed to contribute equal amounts of resources, differential fitness is invariant to the level of l. Because the units of fitness inputs are arbitrary, we can let l define the unit (i.e., $l = 1$), which yields an analytically

[1]This assumption is central to the original polygyny threshold model [e.g., 21, 22] and even its variants [e.g., 23], although some violations have been explored theoretically and empirically [see 24]. A more satisfying model would take into account variation in resources independently held by females. But, as far as we can determine, such a model does not appear analytically tractable in our framework [but see 25, for an alternative approach].

tractable model for male fitness:

$$w_i = n_i \; g_i^{\gamma} \left(\frac{m_i - n_i c}{n_i} \right)^{\mu} \tag{5}$$

When the parameters γ and μ sum to 1—i.e., there are constant returns to scale—each parameter is equal to the fraction of a hypothetical budget that a fitness maximizer would spend to acquire each resource type if the (also hypothetical) price of each resource type were equal to its marginal effect on fitness [18, 20]. When μ is close to 1, the acquisition of additional mates will not lead to fitness increases for a male, because rival resources act as a limiting agent in the production of fitness, with each additional mate's fertility being limited in direct proportion to the share of rival resources she receives. More formally, in the limit as $\mu \to 1$ with $\gamma + \mu = 1$, fitness approaches proportionality to rival resources minus the cost of matting effort: $m_i - n_i c$. Under such conditions, male fitness is maximized by minimizing mating effort and pairing monogamously (at least in reasonable ranges of the parameter space where $m_i > c$). As we see in the main text, when μ is near unity, the population may be monogamous, but inequality in fitness may still be substantial if rival resources are unequally distributed.

3.4 Simulating marriage market outcomes and reproductive skew

We can find the male demand function by noting that:

$$\frac{\partial w_i}{\partial n_i} = g_i^{\gamma} (\frac{m_i - n_i c}{n_i})^{\mu} (1 - \frac{\mu m_i}{m_i - n_i c}) \tag{6}$$

Then, the maximal demand for partners, denoted by d_i, is the number of partners that maximizes a male's fitness, and is given by setting the right-hand side of Eq. 6 equal to 0 and solving for n_i:

$$d_i = \frac{m_i (1 - \mu)}{c} \tag{7}$$

To determine the distribution of females over males, we use a simulation model. First, we create a balanced sample of K males and K females, though it is possible to run the simulation with other sex ratios. In the main text, we assume $K = 100$. We also assume that non-rival resources, g_i, follow an approximately Gaussian shaped Gamma distribution—e.g., $g_i \sim \text{Gamma}(30, 18)$. We then iterate the model over a range of rival resource distributions—i.e., we model $m_i \sim \text{Gamma}(10\phi, \phi)$, where $\phi \in (0.03, 1.64)$. As ϕ shifts over this range, the Gini coefficient—a standard measure of resource inequality—ranges in 0.12 to 0.70. We also consider a range of rival resource elasticity values, $\mu \in (0.12, 0.95)$, and within models, we set the non-rival resource elasticity such that male fitness has constant returns to scale—i.e., $\gamma = 1 - \mu$. Finally, we assume that mating costs scale modestly with rival resource holdings—i.e., we set $c_i = m_i^{0.6}$.

Next, we simulate the mate choice process. First, we calculate male demand for mates according to Eq. 7. Since our simulation must use integral values for the number of partners desired, $\hat{d}_i$, we calculate each male's fitness at both the floor and ceiling of d_i and define $\hat{d}_i$ to be the value which leads to higher fitness. The value $\hat{d}_i$ defines the maximal demand, the number of mates at which the male switches into a state of not seeking addition mates. Until he reaches

that point, we allow each female to choose which male with unsupplied demand to select. It is only upon successful mate-matching that the mating investment cost for a given match is "paid". Thus, polygynous-seeking males do not pay all their mating cost up front, prior to any mate-matching occurring; they keep all of their resources for breeding up until c units of cost is paid at the time of a successful pairing.

To determine female supply, we simulate a voluntary assignment of females over males, in which each female compares her expected fitness under pairing to each and every male in the population who has unsupplied demand. Each female will choose to pair the male with whom she will have maximized fitness. In the ideal-free model, male demand is free to vary as described above. However, in the socially imposed monogamy model, male demand is capped at 1.

After mate matching is complete, inequality in male and female fitness vectors is calculated using the M index, as illustrated in the main text. We iterate the simulation over all combinations of 30 rival resource inequality values and 30 rival resource importance values, for each of the two mate matching algorithms, yielding 1,800 total simulation runs. For details, see the supplemental code included in the GitHub repository: https://github.com/ctross/reproductive_skew.

3.5 Effects of socially imposed monogamy versus ideal-free mate matching on reproductive skew

To generate precise estimates of the effect of socially imposed monogamy on reproductive skew, we compare simulations between mating systems (Fig. 2). Social imposition of monogamy reduces male reproductive skew for low values of rival resource importance and high values of rival resource inequality. Social imposition of monogamy increases female reproductive skew for moderate values of rival resource importance and high values of rival resource inequality. These figures support the qualitative predictions provided in the main text.

4 Polygyny in the Standard Cross-Cultural Sample

Table 2 presents a breakdown of the Standard Polygamy code (v861) [26]. Note that our figures for polyygny are lower than those typically cited from Gray [27], who corrected Murdock's Ethnographic Atlas polygyny codes and found that of the 1,231 sampled societies: 4 were polyandrous, 186 monogamous, 453 showed occasional polygyny, and 588 had frequent polygyny; this has lead to the much cited claim that 85% of human societies are polygynous. It should be noted, however, that Murdock's sample greatly over-represents small scale societies and provides no guarantee of independence of samples. We therefore place more credence in the Standard Cross-Cultural Sample.

5 Reproductive egalitarianism in humans

Our findings suggest a modest—but detectable—degree of reproductive egalitarianism among human males. This may be surprising, given that most human societies allow polygynous marriage and mating [28], and might therefore be expected to show high levels of reproductive inequality, particularly if males can retain multiple wives as they age [29]. Some scholars, for

Table 2: *Standard Polygamy code (v861; from Standard Cross-Cultural Sample). Valid percent refers to the non-missing data.*

	Standard_Polygamy_code_v861	Frequency	Percent	Valid Percent	Cumulative Percent
Valid	Polyandry	2	1.1	1.2	1.2
	Monogamy prescribed	26	14.0	15.1	16.3
	Monogamy preferred, exceptional cases of polygyny	34	18.3	19.8	36.0
	Limited polygyny <20% of married males	51	27.4	29.7	65.7
	Full polygyny 20% or more of married males	59	31.7	34.3	100.0
	Total	172	92.5	100.0	
Missing		14	7.5		
Total		186	100.0		

example, have previously emphasized the extreme levels of reproductive inequality associated with despotism [30], noting consistency with high reproductive skew in animal societies [31]. Such findings, however, may not be representative of the majority of human populations.

Sources of bias towards perceptions of high skew include differential visibility of a few highly successful men [32], strong inter-village variation in skew that makes it difficult to generalize from village-level studies [33], neglect of age structure [34], and differential survival of historical records from despotic societies [35]. On the other hand, acknowledged sampling biases towards household heads or against out-migrants [36] can lead to underestimation of reproductive skew. Ultimately, complete census records of all individuals in a population—from birth to death—provide the least biased estimates of reproductive inequality [37]. Obtaining such detailed data from an unbiased sample of human populations across time and space, however, is no easy feat. The database used in this analysis provides the best-to-date indication of the typical range of reproductive inequality across rural/small-scale human societies, but more detailed data are still needed to test the theoretical models presented here.

6 Data Sources

Tables 3 and 4 present the human and non-human mammal datasets (respectively) used in our analyses. In the case of original (previously unpublished) datasets, citations refer to background information about the populations in question. The proxy measures of fitness used in our study vary: some authors report fertility, others report surviving offspring, others report offspring surviving to a specified age, and still others report number of direct genealogical descendants.

Table 3: *Human datasets included in the main analyses. For any population in which multiple samples are listed, M values were aggregated to reflect the mean sex-specific skew values for that population. We provide example citations, and the names of some ethnographers/principal investigators (PIs) at each site. Fieldwork is always a collaborative affair, and the individuals listed below should be seen as points of contact for research details, not as sole contributors.*

Data sources			
Population	Ethnographers/PIs	Location	Citation
Aché	Hill	Paraguay	[38]
Achuar	Mader	Peru	[39]
Afrobrasilians	Hartung	Brazil	[39]
Afrocolombians [1]	Ross	Colombia	[40]
Afrocolombians [2]	Ross	Colombia	[40]
Agta [1]	Headland and Headland	Philippines	[41]
Agta [2]	Page et al.	Philippines	[42]
Agta [3]	Page et al.	Philippines	[42]
Aka	Hewlett	CAR	[43]
Altiplano [1]	Moya	Peru	[44]
Altiplano [2]	Moya	Peru	[44]
Alyawarra	Denham	Australia	[39]
Ammonni	Bonte	Mauritania	[39]
Apache	Goodwin	USA	[39]
Arsi Oromo [1]	Gibson	Ethiopia	[45]
Arsi Oromo [2]	Gibson	Ethiopia	[45]
Arsi Oromo [3]	Gibson	Ethiopia	[45]
Barí	Beckerman	Venezuela	[46]
BaYaka	Boyette and Lew-Levy	Congo	[47]
Bengali	Shenk	Bangladesh	[48]
Bandongo	Lew-Levy and Boyette	Congo	[47]
Chagga	Caudell	Tanzania	[49]
Chewa	Sear	Malawi	[50]
Choyero [1]	Macfarlan	Mexico	[51]
Choyero [2]	Macfarlan	Mexico	[51]
Chugurpampa	Oths	Peru	[52]
Chuukese	Goodenough	Micronesia	[39]
Cocama	Ruiz	Peru	[39]
Darkhad	Hooper	Mongolia	
Dogon	Cazes	Mali	[39]
Dolgan	Ziker	Siberia	[53]
Efate	Mattison	Vanuatu	
Emberá	Ross	Colombia	[54]
English	Clark	England	[55]
European Royalty	Hamberger	Europe	[39]
Finnish [1]	Lummaa	Finland	[56]

Continuation of Table 3			
Population	Ethnographers/PIs	Location	Citation
Finnish [2]	Helle	Finland	[57]
Gambians	Sear	Gambia	[58]
Hadza [1]	Marlowe	Tanzania	[59]
Hadza [2]	Wood	Tanzania	[60]
Hadza [3]	Blurton Jones	Tanzania	[61]
Hadza [4]	Apicella	Tanzania	[62]
Haiti	DeMarco	Haiti	
Han	Sum	China	[63]
Hental	Grimalda	Papua New Guinea	
Himba	Scelza	Namibia	[64]
Hiwi	Hill	Venezuela	[65]
Interculturales	Pisor	Bolivia	[66]
Inuit	Holmes	Greenland	[39]
Kipsigis	Borgerhoff Mulder	Kenya	[67]
Koore	Caudell	Ethiopia	[68]
Krummhörn	Willfuehr	Germany	[69]
Kung [1]	Howell and Draper	Botswana	[70]
Kung [2]	Marshall	Botswana	[39]
Lamalera	Nolin	Indonesia	[71]
Lapp	Withaker	Scandinavia	[39]
Lovu	Willard	Fiji	[62]
Maasai [1]	Caudell	Tanzania	[72]
Maasai [2]	Caudell	Tanzania	[72]
Madagascar	Golden	Madagascar	[73]
Makushi	Schacht	Guyana	[74]
Malawi	Lanning	Malawi	[75]
Matsigenka	Revilla-Minaya and Bunce	Peru	[76]
Mauritians	Xygalatas	Mauritius	[62]
Maya [1]	Cortez, Pacheco-Cobos, and Winterhalder	Belize	[77]
Maya [2]	Kramer	Mexico	[78]
Maya [3]	Downey	Belize	[79]
Maya [4]	Downey	Belize	[79]
Mayangna	Koster	Nicaragua	[80]
Meriam	Smith, Bliege Bird, and Bird	Australia	[81]
Mestizo [1]	Bunce and Revilla-Minaya	Peru	[82]
Mestizo [2]	Ross	Colombia	[40]
Mestizo [3]	Ross	Colombia	[40]
Miskito	Koster	Nicaragua	[80]
Moseten	Pisor	Bolivia	[83]
Mosuo [1]	Sum	China	[63]
Mosuo [2]	Sum	China	[63]

Continuation of Table 3			
Population	Ethnographers/PIs	Location	Citation
Nganasan	Ziker	Siberia	[53]
Nunamiut	Gubser	USA	[39]
Nyakyusa	McElreath	Tanzania	[84]
Ojibwa	Rogers	Canada	[39]
Pere	Mead	New Guinea	[39]
Pesqueiro	Cohen	Brazil	[62]
Pimbwe	Borgerhoff Mulder	Tanzania	[85]
Polish	Colleran	Poland	[86]
Pumé	Greaves and Kramer	Venezuela	[87]
Pumi	Sum	China	[63]
Saba (Black)	Leslie	Dutch Caribbean	[88]
Saba (White)	Leslie	Dutch Caribbean	[88]
Sainte Catherine	Legrand	Canada	[39]
Sami	Helle	Finland	[89]
Sangu	McElreath	Tanzania	[84]
Semang	Schebesta	Malaysia	[39]
Sena	Mertens	Mozambique	
Shodagor	Starkweather	Bangladesh	[90]
Shuar [1]	Sugiyama	Ecuador	[91]
Shuar [2]	Sugiyama	Ecuador	[91]
Sidama	Caudell	Ethiopia	[92]
Slavey	Helm	Canada	[39]
Sukuma [1]	Borgerhoff Mulder	Tanzania	[93]
Sukuma [2]	McElreath	Tanzania	[84]
Sukuma [3]	Salerno	Tanzania	[94]
Twa	Davis	Angola	
Tamil [1]	Power	India	[95]
Tamil [2]	Power	India	[95]
Tanna [1]	Atkinson	Vanuatu	[62]
Tanna [2]	Atkinson	Vanuatu	[62]
Tanna [3]	Massengill	Vanuatu	
Tikuna	Echeverri	Colombia	[39]
Tiwi	Hart	Australia	[96]
Toba	Valeggia	Argentina	[97]
Torshan	Botte	Mauritania	[39]
Tsimane' [1]	Gurven et al.	Bolivia	[98]
Tsimane' [2]	Godoy et al.	Bolivia	[99]
Turkana [1]	Leslie	Kenya	[100]
Turkana [2]	Leslie	Kenya	[100]
Tuvans [1]	Hooper	Siberia	[101]
Tuvans [2]	Purzycki	Siberia	[62]

Continuation of Table 3			
Population	Ethnographers/PIs	Location	Citation
Waimiri	Silva	Brazil	[39]
Wanindiljaugwa	Rose	Australia	[39]
Warao	Wilbert	Venezuela	[39]
Warusha	Caudell	Tanzania	[72]
Xavante	Salzano	Brazil	[102]
Yasawa	McNamara	Fiji	[62]
Zhuang	Sum	China	[63]

Table 4: *Non-human mammal datasets included in the main analyses. For any species in which multiple samples are listed, M values were aggregated to reflect the mean sex-specific skew values for that species.*

Data sources			
Order	Species	Population	Citation
Artiodactyla	*Capreolus capreolus*	Roe deer	[103]
Artiodactyla	*Cervus elaphus*	Red deer	[104, 105, 8]
Artiodactyla	*Odocoileus virginianus*	White-tailed deer	[106]
Artiodactyla	*Orcinus orca*	Killer whale	[107]
Artiodactyla	*Ovis aries*	Soay sheep	[108, 109]
Artiodactyla	*Ovis canadensis*	Bighorn sheep	[110]
Artiodactyla	*Pecari tajacu*	Collared peccary	[111]
Artiodactyla	*Rupicapra rupicapra*	Northern chamois	[112]
Artiodactyla	*Stenella frontalis*	Atlantic spotted dolphin	[113]
Artiodactyla	*Tursiops truncatus*	Bottlenose dolphin	[114]
Carnivora	*Felis catus*	Feral cat	[115]
Carnivora	*Lycaon pictus*	African wild dog	[116]
Carnivora	*Nasua nasua*	Ringtailed coati	[117]
Carnivora	*Arctocephalus gazella*	Antarctic fur seal	[118]
Carnivora	*Canis rufus*	Red wolf	[119, 8]
Carnivora	*Crocuta crocuta*	Spotted hyena	[120]
Carnivora	*Meles meles*	European badger	[121, 122]
Carnivora	*Mirounga angustirostris*	Elephant seal	[123, 122]
Carnivora	*Panthera leo*	African lion	[124, 125, 8]
Carnivora	*Phoca vitulina*	Harbour seal	[126, 127]
Carnivora	*Suricata suricatta*	Meerkat	[128, 8]
Carnivora	*Ursus americanus*	Black bear	[129]
Carnivora	*Zalophus wollebaeki*	Galapagos sea lion	[130]
Chiroptera	*Cynopterus sphinx*	Indian fruit bat	[131]
Chiroptera	*Myotis bechsteinii*	Bechstein's bat	[132]
Dasyuromorphia	*Antechinus agilis*	Agile antechinus	[133]
Dasyuromorphia	*Antechinus stuartii*	Brown antechinus	[134]

Continuation of Table 4			
Order	Species	Population	Citation
Diprotodontia	*Petrogale penicillata*	Rock wallaby	[135]
Diprotodontia	*Phascolarctos cinereus*	Koala	[136]
Lagomorpha	*Lepus americanus*	Snowshoe hare	[137]
Perissodactyla	*Ceratotherium simum*	White rhinocero	[138]
Perissodactyla	*Diceros bicornis*	Black rhinoceros	[139]
Perissodactyla	*Equus caballus*	Wild horse	[140]
Perissodactyla	*Equus quagga*	Plains zebra	[140]
Pilosa	*Bradypus variegatus*	Brown-throated sloth	[141]
Primata	*Brachyteles hypoxanthus*	Northern muriqui	[142]
Primata	*Cebus capucinus*	White-faced capuchin	[143]
Primata	*Chlorocebus pygerythrus*	Vervet monkey	[144, 122]
Primata	*Gorilla beringei*	Mountain gorilla	[122]
Primata	*Hylobates lar*	Lar gibbon	[145, 8]
Primata	*Leontopithecus rosalia*	Golden lion tamarin	[146]
Primata	*Macaca fuscata*	Japanese macaque	[147, 8]
Primata	*Macaca mulatta*	Rhesus macaque	[148, 149]
Primata	*Macaca nigra*	Crested macaque	[150]
Primata	*Macaca sylvanus*	Barbary macaque	[151, 8]
Primata	*Pan paniscus*	Bonobo	[152, 153, 154]
Primata	*Pan troglodytes*	Common chimpanzee	[155, 156, 157, 153, 8, 158]
Primata	Papio cynocephalus	Savannah baboon	[159, 8]
Primata	*Propithecus verreauxi*	Sifaka	[160, 161, 8]
Proboscidea	*Loxodonta africana*	African elephant	[162]
Rodentia	*Castor fiber*	Eurasian beaver	[163]
Rodentia	*Tamias amoenus*	Yellow-pine chipmunk	[164]
Rodentia	*Tamias striatus*	Eastern chipmunk	[165]
Rodentia	*Fukomys damarensis*	Damaraland mole rat	[166]
Rodentia	*Peromyscus californicus*	California mouse	[167, 8]

Figure 2: *Difference in reproductive skew as a function of socially-imposed monogamy versus mate-matching using the generalized polygyny threshold model introduced by Oh et al. [18]. Male rival resource inequality, R, is measured using the Gini coefficient and ranges $\in (0.12, 0.64)$. Rival resource importance, μ, is measured using the fitness elasticity of rival resources and ranges $\in (0.15, 0.95)$. Non-rival resources, G, are held constant across individuals. Non-rival resource importance, γ, is measured using the fitness elasticity of non-rival resources and is given by the equation $\gamma = 1 - \mu$ to ensure constant returns to scale. Social imposition of monogamy reduces male reproductive skew for low values of rival resource importance and high values of rival resource inequality. Social imposition of monogamy increases female reproductive skew for moderate values of rival resource importance and high values of rival resource inequality.*

(a) *Difference in male reproductive skew between socially-imposed monogamy (SIM) and ideal-free (IFD) mate-matching.*

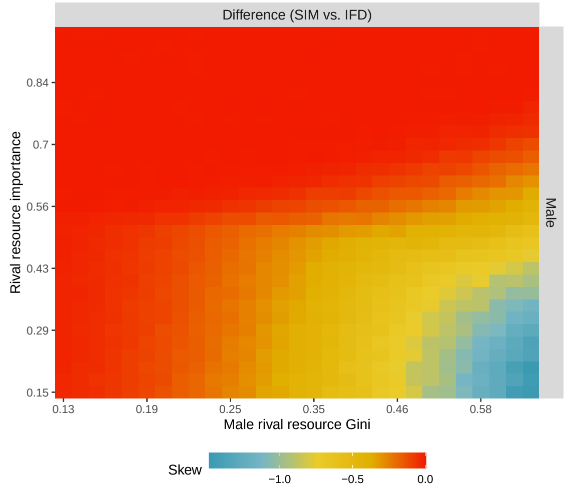

(b) *Difference in female reproductive skew between socially-imposed monogamy (SIM) and ideal-free (IFD) mate-matching.*

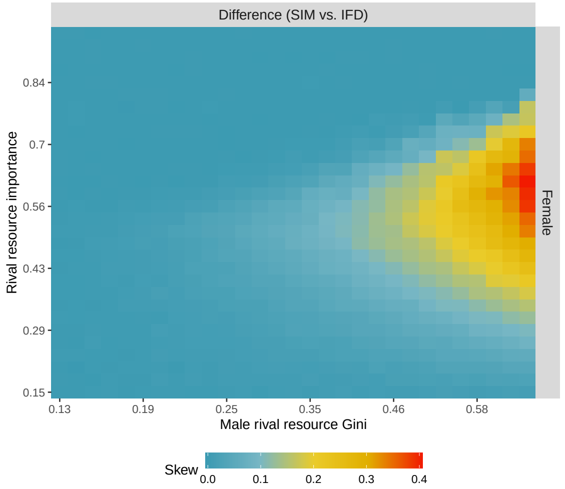

(c) *Difference in sex differences in reproductive skew between socially-imposed monogamy (SIM) and ideal-free (IFD) mate-matching.*

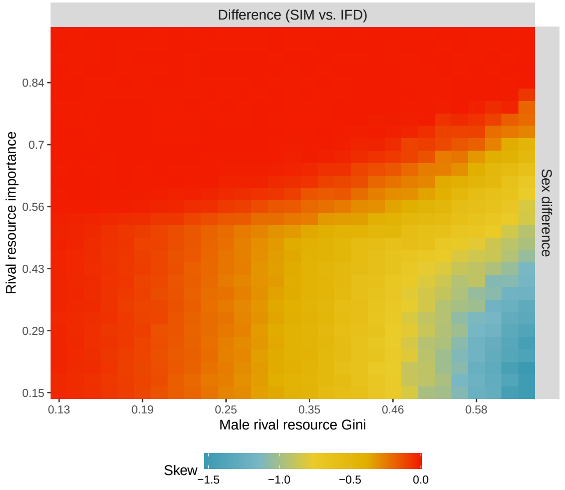

7 Meta-analysis of reproductive skew in mammals

7.1 Model definition

To analyze the patterns of variation in reproductive skew across various mating systems in humans and non-human mammals, we use a Bayesian meta-analysis model. We frame the model in descriptive terms, aiming simply to represent the variation within and between categories (i.e., rather than testing for relationships between predictors and outcomes that are robust to control for phylogentic random effects). We let $\dot{M}_{[n]}$ represent male skew in population n, $\ddot{M}_{[n]}$ represent female skew in population n, and $\hat{M}_{[n]}$ represent the sex difference in skew, $\dot{M}_{[n]} - \ddot{M}_{[n]}$, in population n:

$$\dot{M}_{[n]} \sim \text{Normal}(\dot{\mu}_{[H(n),P(n)]}, \dot{\sigma}_{[H(n)]}) \tag{8}$$

$$\ddot{M}_{[n]} \sim \text{Normal}(\ddot{\mu}_{[H(n),P(n)]}, \ddot{\sigma}_{[H(n)]}) \tag{9}$$

$$\hat{M}_{[n]} \sim \text{Normal}(\hat{\mu}_{[H(n),P(n)]}, \hat{\sigma}_{[H(n)]}) \tag{10}$$

Here, we note that $H(n)$ is an indicator function for if data-point n comes from a human or non-human mammal population, and $P(n)$ is an indicator function for if data-point n comes from a polygynous population. As such, we get estimates of mean skew unique to the interaction of human and polygynous; variance terms are also unique for humans and non-humans.

We use weak priors on the model's mean parameters:

$$\dot{\mu}_{[h,p]} \sim \text{Normal}(0, 5) \tag{11}$$

$$\ddot{\mu}_{[h,p]} \sim \text{Normal}(0, 5) \tag{12}$$

$$\hat{\mu}_{[h,p]} \sim \text{Normal}(0, 5) \tag{13}$$

and standard deviation parameters:

$$\dot{\sigma}_{[h]} \sim \text{Cauchy}(0, 2.5) \tag{14}$$

$$\ddot{\sigma}_{[h]} \sim \text{Cauchy}(0, 2.5) \tag{15}$$

$$\hat{\sigma}_{[h]} \sim \text{Cauchy}(0, 2.5) \tag{16}$$

To estimate mean difference in skew across categories, we calculate contrasts. For example, the contrast in average male skew between polygynous human populations and polygynous non-human populations can be written as:

$$\dot{\delta}_{[p=1]} = \dot{\mu}_{[h=1,p=1]} - \dot{\mu}_{[h=0,p=1]} \tag{17}$$

7.2 Software implementation

Models are fit using Hamiltonian Monte Carlo [168] via the Stan 2.23 C++ library [169]. Models are implemented using an R [170] workflow, through the `rstan` interface. All code used in modeling is included in the supplementary files.

7.3 Model fit: Estimation of M

For each human population, and several non-human mammal populations, we calculated both point estimates of M, and Bayesian posterior estimates of M, using the `SkewCalc` R package and individual-level data. For most non-human mammal data, we did not have individual-level data, and we simply converted published point estimates of B or the coefficient of variation into point estimates of M using the analytical relationships described in Ross et al. [7].

We checked the individual-level data, and the fit of the models to the data, using scatter plots, traceplots, and posterior density plots. Fig. 3 shows an example set of checks using data from a Mestizo population in Colombia. In general, our data-quality checks suggest that there are no obvious data entry errors (e.g., individuals having a reproductive success of -999), that the models fit well, and that both methods of estimating M lead to similar metrics of reproductive skew.

In a few individual-level data-sets, the posterior estimate of M and the point estimate of M diverged appreciably. Accordingly, we visualize both the posterior estimates of M and the point estimates of M in Fig. 3. The correlation of both estimates of M is quite high, $\rho = 0.91$ for males and $\rho = 0.9$ for females. As such, we simply use the posterior median estimate of M in the main analyses.

7.4 Model fit: Comparative models

Model fit was assessed in the main analysis using standard metrics like $\hat{r}$, effective samples, and traceplots. All indicators suggest good mixing, and convergence of multiple chains to the same region of high posterior density. See Fig. 4.

8 Robustness check

To test if our results are robust to dropping populations with small samples, or less rigorous demographic protocols, we repeat our main analysis, including only 29 populations—the subset for which sample size was large and the data were collected for the purposes of demographic analyses. Figs. 5–7 show the results of replicating our main analysis using this restricted sample of human populations. All of our qualitative findings hold, though the numerical values of estimates shift slightly.

Figure 3: *Data and model checks for an example human population (a Mestizo population from Colombia).*

(a) *Scatterplot of male reproductive success on age. A smoothing spline is superimposed on the data.*

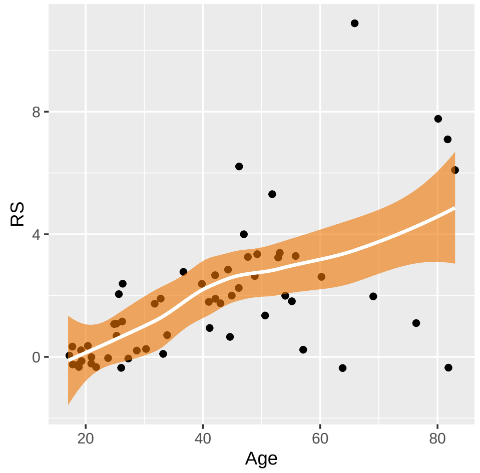

(b) *Posterior density of male M (orange curve, with 90% credible interval shaded). The posterior median is plotted as a white vertical bar, and the point estimate of M is plotted as a dark orange vertical bar.*

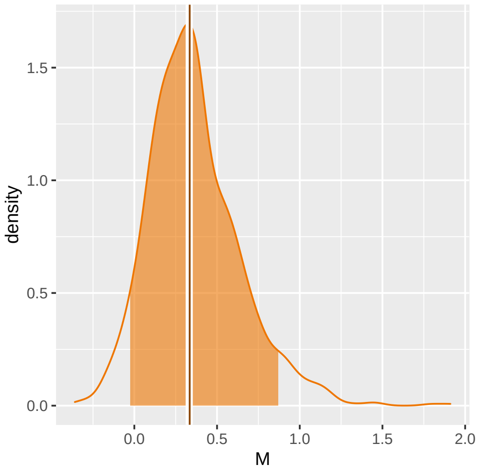

(c) *Traceplot of the parameters from the Bayesian model of M. The chains show stationarity and good mixing.*

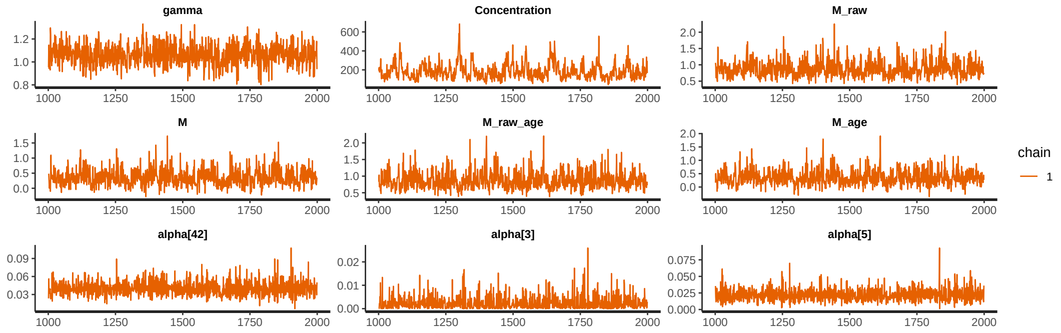

Figure 3: *M values by population in humans. Posterior 90% credible intervals are plotted as black bars. Posterior medians are plotted as black points. Point estimates of the M index are plotted as red points. Both methods of estimating M lead to similar measures of skew: the correlation between posterior median estimates of M and point estimates of M is:* $\rho = 0.91$ *for males and* $\rho = 0.9$ *for females.*

(a) *Male M values in humans.*

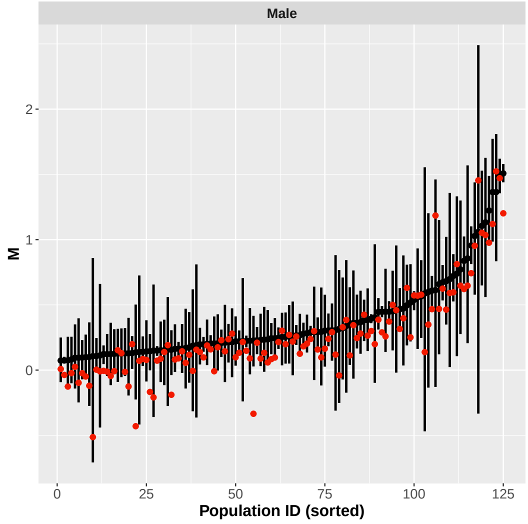

(b) *Female M values in humans.*

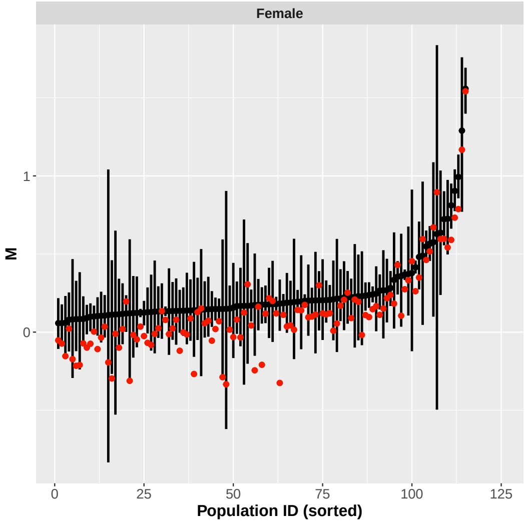

(c) *Scatter of all posterior median and point estimates of M. The dashed red line shows exact equality. The blue line is the linear best fit line. Both estimating procedures yield highly correlated outcomes, but the Bayesian model is suspicious of negative skew values.*

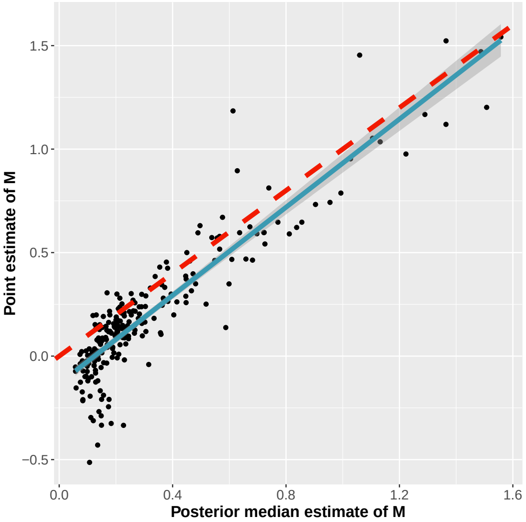

Figure 4: *Traceplots of all model parameters in the main comparative analyses. The chains show stationarity and good mixing.*

(a) *Model 1a, humans versus non-human mammals*

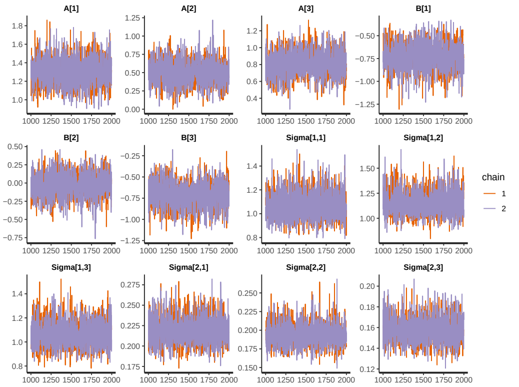

(b) *Model 1b, humans versus non-human primates*

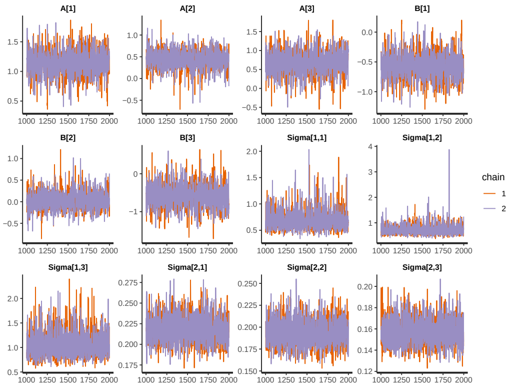

(c) *Model 2a, polygyny×human interaction*

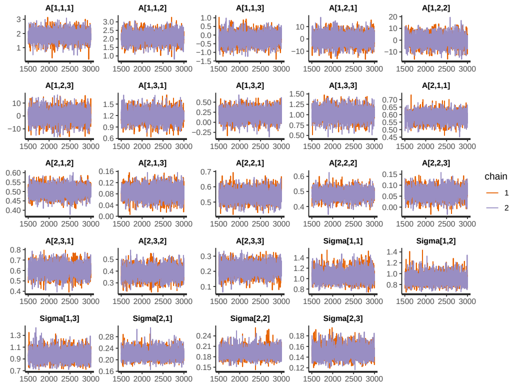

(d) *Model 3a, polygynous humans versus polygynous non-human mammals*

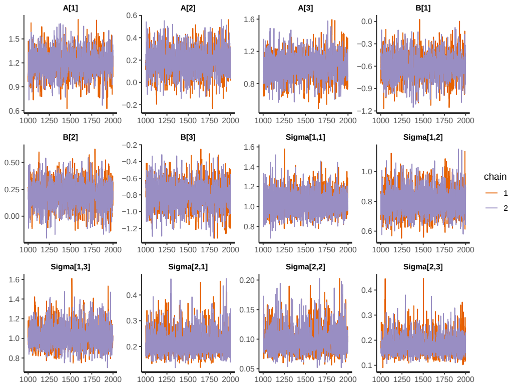

(e) *Model 3b, polygynous humans versus polygynous non-human primates*

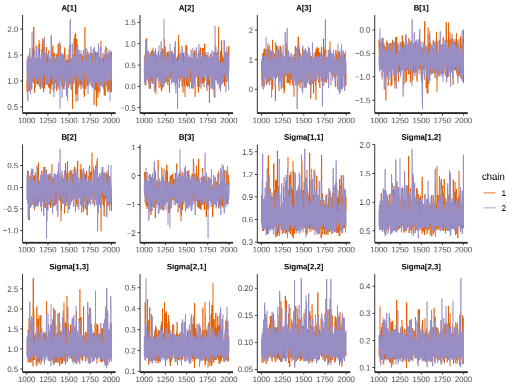

Figure 5: *Posterior distributions of the difference in reproductive skew between humans and non-human mammals/non-human primates. Points represent posterior mean differences, and horizontal bars represent 89% credible regions. The dashed vertical line at zero indicates no difference. Humans stand out from both non-human mammals, generally, and non-human primates, specifically, in terms of having lower values of average male reproductive skew and lower sex differences in skew. Female reproductive skew, however, appears similar in humans and both non-human mammals and non-human primates—on average. Sample sizes:* $N = 29$ *human populations,* $N = 49$ *non-human mammal species,* $N = 12$ *non-human primate species.*

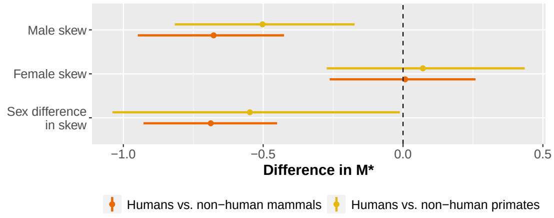

Figure 6: *Posterior distributions of reproductive skew values* (M^*) *in humans as a function of marriage system. Points represent posterior means, and lines represent 89% credible regions. The dashed vertical line at* $M^* = 0$ *indicates that reproduction is neither positively skewed, nor more equal than would be expected by a random model. In general, male reproductive skew appears fairly invariant to marriage system. Female skew appears slightly higher in human populations with socially imposed monogamy (*Normative monogamy*) than populations in which polygyny is widely practiced (*Normative polygyny*). Across all marriage system types, sex differences in skew are reliably different from zero—indicating that male reproduction is slightly more unequal than female reproduction, even where monogamy in imposed (*Normative monogamy*) or frequent (*Polygyny rare, but tolerated*). In contexts where polygyny is common, sex difference in skew are especially high. Sample sizes:* $N = 29$ *human populations* (17 *normative monogamy,* 7 *polygyny permitted,* 5 *normative polygyny).*

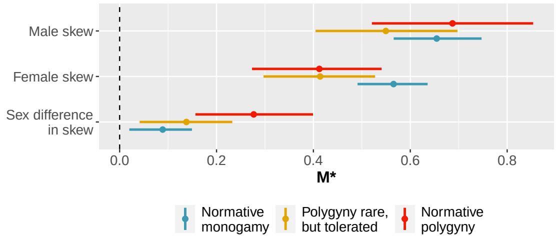

Figure 7: *A different patterning of skew in polygynous human populations and polygynous non-human mammals. Points represent posterior means, and lines represent 89% credible regions. The dashed vertical line at* $M^* = 0$ *indicates that reproduction is neither positively skewed, nor more equal than would be expected by a random model. Male reproductive skew in polygynous humans is substantially lower than in polygynous non-human mammals. Female skew is also slightly higher in polygynous human populations than in polygynous non-human mammals. Sex differences in skew are therefore much lower in polygynous human populations than in polygynous non-human mammals. Sample sizes:* $N = 5$ *polygynous human populations and* $N = 41$ *polygynous non-human mammal species.*

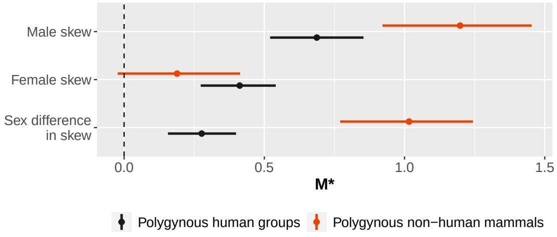

9 Extended Acknowledgments

We express our gratitude to all of the people who chose to take part in this study, and all of the research assistants, translators, and colleagues who help to collect and process the data.

Thanks to Geoff Wild, Christoph Hauert, Woodrow Denham, Martin Surbeck, Kevin Langergraber, Joseph Henrich, Robert Boyd, Kay E. Holekamp, Russell Van Horn, Eli Swanson, Dieter Lukas, Peter Nonacs, Jane Lancaster, and Mateus Dapitaia for helpful input, feedback, and/or translation. This work was conducted as a part of the Working Group: "Emergence of Hierarchy and Leadership in Mammalian Societies" at the National Institute for Mathematical and Biological Synthesis, supported by NSF Award DBI-1300426 and the University of Tennessee, Knoxville. It was also supported by NSF awards SMA-1329089 and SMA-1743019, and the Santa Fe Institute, as well as the Max Planck Institute for Evolutionary Anthropology, Department of Human Behavior, Ecology and Culture. SG was supported by the U.S. Army Research Office grants W911NF-14-1-0637, W911NF-17-1-0150, and the Office of Naval Research grant W911NF-18-1-0138. Additional funding for data collection was provided by the Wenner-Gren Foundation for Anthropological Research awards: 8913 and 7970, by NSF awards: BCS-0924630, BCS-0925910, BCS-0848360, BCS-0514559, BCS-0613226, BCS-0827277, SES-9870429, and DDRIG-1357209, by the National Geographic Society awards: HJ-099R-17, 20113909, 8671-09, and 7968-06, by the Kone Foundation awards: 086809, 088423, and 088423, and by the Jacobs Foundation, the UCSB Broom Center for Demography, and the UCSB Department of Anthropology.

Lomas Barbudal Monkey Project field assistant acknowledgments: S. Perry and I. Godoy thank the following field assistants who each contributed more than 1 year to data collection during 1990-2012: B. Barrett, L. Beaudrot, M. Bergstrom, R. Berl, A. Bjorkman, L. Blankenship, T. Borcuch, J. Broesch, J. Butler, F. Campos, C. Carlson, M. Corrales, C. deRango, C. Dillis, N. Donati, G. Dower, R. Dower, K. Feilen, K. Fisher, A. Fuentes J., M. Fuentes, C. Gault, I. Gottlieb, J. Gros-Louis, L. Hack, S. Herbert, C. Hirsch, C. Holman, S. Hyde, E. Johnson, L. Johnson, M. Kay, D. Kerhoas-Essens, S. Kessler, S. Lee, S. Leinwand, T. Lord, S. MacCarter, J. Manson, W. Meno, C. Mitchell, Y. Namba, A. Neyer, C. O'Connell, J.C. Ordoñez J., N. Parker, B. Pav, R. Popa, K. Potter, K. Ratliff, H. Ruffler, S. Sanford, C.M. Saul, I. Schamberg, A. Scott, C. Schmitt, J. Verge, A. Walker Bolton, E. Wikberg, E. Williams. And they extend special thanks to the two long-term field site managers, W. Lammers, and H. Gilkenson, whose aid in training new field assistants was so critical to maintaining the accuracy of the long-term demographic data. The Lomas Barbudal Monkey Project acknowledges the funding sources which enabled the collection of these data on wild Capuchin demographics: NSF grants (BCS-0848360, BCS-0613226, and SES-9870429), a postdoctoral fellowship (9633991) and a graduate research fellowship; National Geographic Society grants (20113909, 8671-09, 7968-06 and a 1991 grant to S. Perry and B. Smuts); a grant from the Wenner-Gren Foundation for Anthropological Research; and six LSB Leakey Foundation grants. The Max Planck Institute for Evolutionary Anthropology, the University of Michigan, and UCLA provided additional funding and small grants. I. Godoy was funded by NSF dissertation improvement grant (to I. Godoy and S. Perry, BCS-1232371), a Leakey Foundation grant (20120195), a Wenner-Gren grant (443831), a grant from the International Society for Human Ethology, and two small internal grants from UCLA.

10 Statements regarding research approvals/permissions

Our analyses draw on data from different sources, including: (i) published estimates (i.e. summary statistics of mean and variance, or other compatible measures of skew), (ii) publicly-available published data-sets, and (iii) data-sets that were collected in the course of prior research by co-authoring researchers. No human subjects data were collected prospectively for the current study; however, co-authors were responsible for obtaining informed consent from research participants at the time data were collected, and were responsible for ensuring that their protocols were approved (as needed) by the relevant IRB boards, local authorities, and community leaders. Site-specific information for each site described in Table 3 is provided below. Because our datasets are based on surveys/interviews and do not include information beyond the anonymized age, sex, and number of offspring of adult residents, the methods do not pose a risk to the privacy or confidentiality of research participants.

10.1 Data from published sources

Kinsources database A large number of data-sets were taken from the publicly available *Kinsources* database. Datasets are published in *Kinsources* only if they meet the requirements of internal coherence, sufficient documentation, and conformity to privacy protection. The submission process is supervised by a scientific committee composed of international experts from anthropology, history, and social network analysis.

Here, we used data from the following data-sets: Achuar, Afrobrasilian, Alyawarra, Ammonni, Apache, Chuukese, Cocama, Dogon, European royalty, Inuit, Kung, Lapp, Nunamiut, Ojibwa, Pere, Sainte Catherin, Semang, Slavey, Tikuna, Tiwi, Torsan, Waimiri, Wanindiljaugwa, Warao. No IRB approvals were sought for use of these data, as they are anonymized, publicly available data-sets that have been previously reviewed by the *Kinsources* scientific committee.

Xavante; Brasil (Xavante) Data were taken from the paper: "Further Studies on the Xavante Indians" by Xavante et al. (1967): "Table 8. Surviving Offspring In Completed Families".

No IRB approvals were sought for use of these data, as they are an anonymized, publicly available data-set. The original authors acknowledge: "Serviço de Proteção aos Indios for the necessary authorizations and facilities".

Maya; Mexico (Kramer) According to Hackman and Kramer (2021) in *Kin ties and market integration in the Mayan Yucatan* in *Social Sciences, special issue on the Behavioral Ecology of the Family* "The study was conducted according to the guidelines of the Declaration of Helsinki, and approved by the Institutional Review Board of the University of Utah (#00093510)".

Pumé; Mexico (Greaves and Kramer) According to Kramer, Schacht, and Bell (2017) in *Adult sex ratios and partner scarcity among hunter-gatherers: implications for dispersal patterns and the evolution of human sociality* in *Philosophical Transactions of the Royal Society B*, censuses and reproductive histories were collected by Greaves and Kramer in 1990, 1992, 1993 and 2005–2007. Interviews were conducted in the Pumé language. The field research performed by K.L.K. was approved by the Institutional Review Board at Harvard University F15718-102.

Tsimane' database; Bolivia (Godoy et al.) We acknowledge the Program of Cultural and Biological Anthropology of the National Science Foundation (NSF) in the USA and TAPS for making the data publicly available. Godoy and colleage state: "To begin the research we received approval from the Tsimane' governing body, the Tsimane' Council, and from the IRB offices of USA universities managing the research grants". No IRB approvals were sought for use of these data, as they are an anonymized, publicly available data-set.

!Kung database (Howell and Draper) We acknowledge the University of Toronto T-Space program for making the data ("handle/1807/10395") publicly available. Howell states in "Demography of the Dobe !Kung, Aldine de Gruyter, New York": "I acknowledge the contributions to this work by the government of Botswana, which gave research permission and logistic support". No IRB approvals were sought for use of these data, as they are an anonymized, publicly available data-set.

Agta database; Philippines (Headland and Headland) We acknowledge SIL for making the Agta Demographic Database (see https://www.sil.org/resources/archives/9299) publicly available. Headland provides full IRB and informed consent documentation in the database archives. No IRB approvals were sought by us for use of these data, as they are a publicly available data-set.

Krummhörn database, Germany (Voland and Willfuehr) The Krummhörn database (see https://search.gesis.org/research_data/ZA8630) contains demographic data on families who lived in 13 north-west German parishes during the 18th and 19th centuries. Data were compiled based on review of public records, not human subjects research. IRB approvals were not sought by us for use of these data, as they are a publicly available data-set.

English database; England (Clark) The records from Clark's English database come from deceased individuals 1858–2012 , and are based on records at the Principal Probate Registry in London. IRB approvals were not sought by us for use of these data, as they are based on review of public records, not human subjects research.

Sami and Finnish database; Finland (Helle and Lummaa) Helle and Lummaa compiled demographic data from several seventeenth to nineteenth century populations of Northern Scandinavia from Finnish parish registers. IRB approvals were not sought by us for use of these data, as they are based on review of public records, not human subjects research.

Gambian database; Gambia (Sear and McGregor) The data were collected from four villages in rural Gambia continuously from 1950–1980 by Ian McGregor, under the auspices of the U.K. Medical Research Council. No IRB approvals were sought for use of these data, as they are an anonymized, previously published data-set.

10.2 Data from co-authoring researchers

Ache; Paraguay (Hill et al.) The Ache data included here were collected from 1979–1996, and then analyzed over a period of another 15 years. 17 different data collection IRB approvals were granted by the University of Utah, Emory University, the University of Michigan, the University of New Mexico, and subsequent data analysis IRBs were obtained from Arizona State University each year from 2006 to 2023. During the periods of data collection, no Paraguayan National research permits were required; instead we obtained consent directly from the community chiefs and representatives. All data were deidentified more than 20 years ago and all data results were discussed with the community prior to the publication of the 1996 book "Ache Life History". The Ache community leaders repeatedly reinvited us to work in their communities during the study period (Hill has worked with the Ache every year since 1977). Research goals and results were frequently discussed with the community. In later years, signed consent forms were obtained from each interview informant. Verbal consent and interviews were obtained directly in the Ache language.

Afrocolombians, Emberá and Mestizos; Colombia (Ross) The Colombian data included here were collected by CR and local research assistants as part of a wider, longitudinal field study on wealth inequality, demography, and social network structure. Informed consent was obtained from each respondent prior to data collection, and from the community leader or local community council, when appropriate. Due to limited literacy rates, informed consent was obtained verbally after providing respondents with a verbal description (in Spanish) of the research process and explaining how their data will be used (anonymously, for research purposes); in addition, all participants were provided with a written consent document for their own reference. All field protocols were approved by the Department of Human Behavior, Ecology, and Culture at the Max Planck Institute for Evolutionary Anthropology in Leipzig, Germany.

Agta; Philippines (Page et al.) For all data collection (2013–2014) included in the wider project on demography, social support and health, ethical approval was provided by the UCL Ethics Committee (UCL Ethics code 3086/003). Research was carried out with permission from local government and tribal leaders in Palanan. Informed consent was obtained from all participants, and parents signed the informed consents for their children (after group and individual consultation, and explanation of the research objectives in the Agta language).

Aka; Central African Republic (Hewlett) The study and data collection methods were approved by the Institutional Review Board at Southern Oregon State University and the Haut Commissaire de la Recherche Scientifique et Technologique in the Central African Republic. At the regional level, the study was presented to and approved by Mbaiki and Moboma authorities and community leaders, and—at the local level—village and camp members individually provided verbal informed consent before data were collected.

Altiplano; Peru (Moya) Demographic data from Quechua and Aymara speakers were collected as part of a broader ethnographic project with UCLA IRB exemption (#08-169). They were

collected from a rural town and two nearby communities. The project was introduced in community meetings at each of the 3 sites, and at each, the community agreed to allow individuals to participate. Each participant additionally gave verbal consent before each interview either in Spanish if bilingual, or their native language if not (with the help of translators).

Arsi Oromo; Ethiopia (Gibson) Research permission and ethical approval were granted by the Research and Ethics Committee at Addis Ababa University and regional governmental authorities in Ethiopia. Data collection methods were also reviewed and approved by the University of Bristol Research and Ethics Committee. Surveys were administered in 2016 and 2017 by local field assistants fluent in Oromiffa. Informed consent was obtained from each participant in Oromiffa, either by obtaining a signature or thumb print, prior to the survey being administered.

Barí; Venezuela (Beckerman and Lizarralde) Data collection methods were approved by the Institutional Review Board of the Pennsylvania State University. Following the protocol initiated by Lizarralde in 1961, and familiar to all informants, informed consent from all individuals was obtained verbally prior to data collection.

BaYaka and Bandongo; Republic of the Congo (Boyette and Lew-Levy) Initial permission to conduct research in the village was given by the village council during a community meeting with A.H.B. in 2015. Subsequently, permission was granted each year after a community meeting during which that year's work was explained. Individual verbal consent was received from all participants upon recruitment for the study, subsequent to the community meeting. The verbal consent process, as well as all data collections and methods, were conducted based on Duke University and University of Notre Dame ethics guidelines. Permission to conduct the research in the Republic of the Congo was granted by the Centre de Recherche et D'Etudes en Sciences Sociales et Humaines and was supervised by the Institut National de Recherche en Sciences Exactes et Naturelles. The Institutional Review Boards of Duke University (Protocol no. 2017-0038), the University of Notre Dame (no. 18-02-4397), and the University of Cambridge Department of Psychology Research Ethics Committee (PRE.2018.023) also approved the research.

Bengali; Bangladesh (Shenk) All data were collected in accordance with human subjects research protocols approved by the Institutional Review Boards at both the University of Missouri and the International Centre for Diarrhoeal Disease Research, Bangladesh (ICDDR,B). All data were collected via interviews in Bengali, and all research participants gave informed consent prior to their inclusion in the study.

Chagga, Warusha, Sidama, Koore, and Maasai; Tanzania and Ethiopia (Caudell) Data collection methods in Tanzania and Ethiopia were approved by the University of Cincinnati IRB (#00003152). In Tanzania, additional approvals were attained from the Medical Research Coordinating Committee of the National Institute for Medical Research in Tanzania (#NIMR/HQ/R.8a/Vol.IX/2926) and district officials. In Ethiopia, additional approvals were obtained from Hawassa University (ET/HW/#3765) and kebele officials. In both countries,

informed consent was obtained from each individual after explaining the form in Kiswahili/Kimaa (Tanzania) or Amharic/Korette (Ethiopia) and prior to any data collection. For those who could not provide a signature, thumbprints were requested.

Chewa; Malawi (Sear) Data collection for this site was completed long before Institutional Review Board assessment was required. However, following local norms, the project was described at a community meeting and approved by community leaders. Subsequently, informed consent from individuals was obtained verbally prior to data collection

Choyeros; Mexico (Macfarlan) Permission to conduct this research was obtained through the University of Utah Institutional Review Board (IRB #00083096), as well, through signed written agreements with official representatives ("subdelegados") from each community. In accordance with each oversight body, consent was obtained from all heads of household to conduct research, and was recorded by the lead investigator (SJM) at the time of the interview. Because not all participants could read or write, consent to participate was established verbally following a description of the project in Spanish.

Chugurpampa; Peru (Oths) Data collection methods were approved by the Institutional Review Board at the University of Alabama (IRB #12-OR-199-R7). Permission to conduct research was provided in letter form by the Department of Anthropology of the Catholic University of Peru, as well as by a public vote of the community association of Chugurpampa. Informed consent was obtained verbally after providing respondents with a written document, as well as a verbal explanation (in Spanish) of the research process and of how their data will be used (anonymously, for research purposes).

Darkhad and Tuva; Mongolia and Siberia (Hooper et al.) Data collection methods were approved by the Institutional Review Board at Emory University (Emory IRB #82568) and the National Museum of Mongolia. Permission for research was established through Tuvan State University, the Russian Academy of Sciences, and the National Museum of Mongolia. Informed consent was established with participants with the assistance of multilingual translators.

Dolgan and Nganasan; Siberia (Ziker) The data from northern Russia included here were collected by John Ziker. Consultation with the community leader and the regional association of Indigenous people occurred prior to initiating the research. Informed consent was obtained from each respondent prior to data collection. Following local standards at the time, informed consent was obtained verbally after providing respondents with a verbal description (in Russian) of the research process and explaining how their data will be used (anonymously, for research purposes). Data collection protocols were approved by the Institutional Review Boards at Boise State University (2007), Emory University (2003 with J. Henrich), and University of Alaska Fairbanks (2001).

Hadza; Tanzania (Wood) The Hadza data included here were collected by BW and local research assistants as part of a wider, longitudinal field study on subsistence and social organization

between 2004 and 2014. All permits were approved by the Tanzanian Commission for Science and Technology (COSTECH Permit number 2014-146-ER-2000-80, entitled The Behavioural Ecology of Foraging in the Lake Eyasi Area). Regional, district, and local community leaders provided approval, and informed consent was obtained verbally from each respondent prior to data collection in Kiswahili, due to limited literacy rates. All procedures were approved by Yale University IRB 1302011517.

Hadza; Tanzania (Blurton Jones) Hadza field research cited here was approved by the UCLA Human Subjects Protection Committee (#G95-06-106 and onward), and the Tanzania National Scientific Research Council, renewed or re-awarded for each visit from 1985 to 2000.

Hetal; Papua New Guinea (Grimalda) Data from Hetal, Bougainville, PNG, were collected by G.G. and two local research assistants within the project "Social networks, Norms and Patterns of Cooperation". The study received ethical approval by the Kiel Institute for the World Economy Ethics Committee (EP-1-2018) and was reviewed and approved by both the PNG National Research Institute and the Bougainville Regional Government. Informed consent was obtained by the community leaders and from each respondent prior to data collection, after the study was explained in public meetings. All participants had access to the study information sheet, which was read aloud in Tok Pidgin (or in a local language if necessary) by members of the research team. A copy of the information sheet was made available to participants upon request. Participants signed the informed consent or gave verbal approval if illiterate.

Himba; Namibia (Scelza et al.) The Himba data included here were collected by Brooke Scelza and Sean Prall, with a team of local research assistants, as part of long-term study of marriage and family dynamics. The study was approved by the UCLA Institutional Review Board (#10-000238). Research visas were granted by the Namibian Department of Home Affairs, with support from the University of Namibia. Support and approval for the project was obtained from the Chief of Omuhonga each year of data collection, in addition to individual-level verbal consent from all adults.

Hiwi; Venezuela (Hill et al.) The Hiwi data included here were collected between 1985 and 1991. Local research permits were obtained from Departmento de Asuntos Indigenas, in San Fernando de Apure. IRB approval was given by the University of Utah, Emory University, and the University of Michigan. All data were deidentified more than 20 years ago. The local community leaders repeatedly invited us to return and approved all research plans. Verbal consent and interviews were obtained directly in the Hiwi language.

Interculturales and Moseten; Bolivia (Pisor) Participants were interviewed by ACP and two research assistants in April–June 2017. Given mixed literacy, but familiarity with signing forms, participants were read a consent form and provided their written consent via signature. Study protocols were approved by the communities, the Mosetén Tribe (Organización del Pueblo Indígena Mosetén), and the Department of Human Behavior, Ecology, and Culture at the Max Planck Institute for Evolutionary Anthropology in Leipzig, Germany.

Kipsigis, Pimbwe, and Sukuma; Kenya and Tanzania (Borgerhoff Mulder) The Kipsigis data included here were collected by M.B.M. between 1981 and 1984 under annual permits from the Office of the President, Nairobi, Kenya. At the time of data collection, institutional review processes for demographic data collection were not in place at the researcher's home institution. Individuals were nevertheless included in the sample only if they volunteered to participate in the study. All data are anonymized. The Pimbwe and Sukuma data included here were collected by M.B.M. and local research assistants as part of a wider, longitudinal field study on wealth inequality, demography, child health, and polygyny between 1995 and 2016. All permits were approved by the Tanzanian Commission for Science and Technology (COSTECH Permit number 2011-181-ER-2007-67, entitled "Development, Health, Natural Resource Use and Demography: Continuation of Miscellaneous Projects in Rukwa and Beyond"). Regional, district, and local community leaders provided approval. Informed consent was obtained verbally from each respondent prior to data collection in Ki-Swahili, due to limited literacy rates. All procedures were approved by The University of California at Davis, IRB protocol 436682.

Lamalera; Indonesia (Nolin) The Lamalera data included here were collected in 2006 as part of field research reviewed and approved by the Institutional Review Board, Human Subjects Division, of the University of Washington (#04-4847-G 01), and with permission from the Indonesian National Institute of Sciences (Lembaga Ilmu Pengetahuan Indonesia), with the cooperation and support of research counterpart Dr. Dedi Adhuri (LIPI). Informed oral consent was secured in Indonesian by the investigator from all participants. Funding for this research was provided by the National Science Foundation (USA) grant BCS-0514559.

Lovu; Fiji (Willard) Data included here were collected by A.W. and local research assistants as part of the Cultural Evolution of Religion Consortium's Evolution of Religion and Morality Project. Due to variable literacy rates, all participants were provided with a verbal description of research process, the anonymity of the data collected, and how their data would be used in Fiji-Hindi (the local language). Informed consent was given verbally by all participants and recorded by the research assistants. Participants were further offered a written document with the same information they were given verbally for their records if they wanted it. All protocols were approved by the Institutional Review Board at University of British Columbia (BREB #H13-00671).

Madagascar; Madagascar (Vaitla and Golden) All methods were carried out in accordance with relevant guidelines and regulations. Household recruitment and enrollment and individual consent or assent procedures followed our IRB approved studies (Protocols #21-0692 and #15–2230, Committee on the Use of Human Subjects, Office of Human Research Administration at the Harvard T.H. Chan School of Public Health). Consent forms were read by literate study members, and our team read the form contents to illiterate study members. After a discussion of the study materials, participants were invited to participate in the study. Informed consent was obtained from adults. Informed consent was obtained from a parent and/or legal guardian for minors/children below 18 years of age. Verbal assent was obtained from children over 12 years of age, and permission was obtained from parents or guardians of younger

children. The study was also reviewed and approved by the Malagasy Ministry of Health (MOH), the ethical committee of the Institut National de Santé Publique et Communitaire (INSPC) No 03/MSANP/SG/INSPC/DG/DFR in Madagascar, district medical inspectors, and local community leaders (e.g., Président Fokontany). Study subjects were not compensated for their participation. However, the population did receive benefits for participating by having access to healthcare professionals working in their community.

Makushi; Guyana (Schacht) Data collection methods were approved by The University of California at Davis IRB (17795-1). Permission to perform research was also given by the Ministry of Amerindian Affairs in Guyana and community leaders. Informed consent was obtained from each respondent prior to data collection.

Matsigenka and Mestizo; Peru (Revilla Minaya and Bunce) The data presented here were collected in 2012–2014 as part of larger projects exploring Matsigenka environmental perceptions and cultural norm dynamics among Matsigenka and Mestizos. Due to limited literacy, verbal informed consent was obtained by the researchers from each participant in her or his native language (Matsigenka or Spanish), after explaining the purpose of the research and protections of participant identity. Following local governance norms, prior to individual-level data collection in the Matsigenka communities, we also obtained communal informed consent during community assemblies. All research protocols were approved by the Institutional Review Boards of Vanderbilt University (IRB #110531) and the University of California, Davis (Protocol No. 226284-2), and authorized by the Peruvian Servicio Nacional de Áreas Naturales Protegidas por el Estado (SERNANP).

Mauritius; Mauritius (Xygalatas) The Mauritian data were collected in two waves, in 2013 and 2015. The study was approved by the Ethical Review Board of the Czech Association for the Study of Religions. Before the second wave, D.X. moved to the University of Connecticut (UConn), additional permission was obtained by UConn's Institutional Review Board. All participants provided written consent in the local Creole language, and all materials were administered by local research assistants.

Maya, Belize (Downey) The Belize data were collected as part of a larger, long-term research project into the social and ecological dynamics of land-use. Data collection methods were reviewed and approved by the Ohio State University Institutional Review Board (OSU IRB #2017B0387), and the Institute for Social and Cultural Research at the Belize National Institute of Culture and History (NICH Permit No. ISCR/H/2/68). Fieldwork was conducted after receiving permission from the alcalde and Chairman in the study villages. Due to limited literacy rates, informed consent was obtained verbally from each respondent with the assistance of multilingual translators prior to data collection.

Maya; Mexico (Pachecho-Cobos, Cortez, and Winterhalder) The Institute for Social and Cultural Research (ISCR) issued Carmen Cortez, Luis Pacheco-Cobos, and colleagues research permits (ISCR/H/2/6 and ISCR/H/2/5) to conduct research during 2011–2013 under the auspices

of the National Institute of Culture and History (NICH). Local authorities verbally agreed with researchers' plans, and authorized them to live in the village, walk freely, and participate in residents' activities whenever such residents agreed for them to do so. All individuals provided informed consent before participating in surveys. Consent was obtained verbally with household heads while running the Household Demographic Survey. Each member of the research team, upon arrival in the field, also got permission from the community's leaders to conduct research.

Mayangna and Miskito; Nicaragua (Koster) Data collection methods were approved by the Institutional Review Board at the University of Cincinnati (2015-9167). Following local norms, the project was described at a community meeting. Subsequently, informed consent from individuals was obtained verbally prior to data collection.

Meriam; Australia (Smith, Bliege Bird, and Bird) Approval to conduct this study was granted by the Mer Island Community Council in 1995. Following NSF awards to E.A.S. (w/ R.B.B. and D.W.B. as postdocs), approval was obtained from the University of Washington IRB in 1996. Research was conducted over a 3-year period (1997–1999). An oral consent form was employed for all interviews, as well as observational data. All participants were informed that the information we collected was coded and their identities in all records kept private. At the outset of the study, participants were assigned identity codes, with links to names known only to the researchers.

Mosuo, Zhuang, Pumi, and Han; China (Sum, Mattison, et al.) Data were collected in 2017 by Chun-Yi Sum, under protocols approved by the University of New Mexico's IRB (06915) with additional ethical review by Fudan University (16268). Voluntary informed consent was established via verbal description of the project in Mandarin Chinese, translated as necessary into the local dialect or Mosuo language (Naru) with the assistance of a local guide. Participants were provided a copy of the project information sheet and given opportunity to ask questions or withdraw from the study without penalty.

Pesquiero; Brazil (Cohen) Participants were randomly selected from a complete register of all adults in the village and invited to take part in the study. Informed consent was obtained prior to participation. A local research assistant provided information about the study, data confidentiality and use, and voluntary withdrawal to the group verbally (in Portuguese) and participants signed a written consent form. Data collection methods were approved by the ethical review board of the School of Anthropology and Museum Ethnography, University of Oxford (SAME/CUREC 1A 13-50).

Polish; Poland (Colleran) Data were collected between 2009–2010 as part of an anthropological-demographic project, approved by the Ethics Committee of the Department of Anthropology at University College London (UCL). Study aims and protocols were explained first to community leaders (in Polish) who themselves made a public announcement in local Churches prior to data collection in a particular village. Individual participants were provided with written documentation about the project aims and protocols and signed written consent forms before participating

in interviews. All documentation and consent discussions were provided / carried out in Polish by H.C. or by a local field assistant.

Saba and Turkana; Dutch Caribbean and Kenya (Leslie) The Turkana data utilized here were collected as part of a wider, long-term multidisciplinary study (the South Turkana Ecosystem Project) of the regional ecosystem in Turkana District, Kenya, between 1982 and 1996. Data collection methods were approved by the Institutional Review Boards of the State University of New York at Binghamton (1982–1992) and the University of North Carolina at Chapel Hill (1992–1996), following obtainment annual research permits from the Office of the President, Nairobi, Kenya. Informed consent was obtained verbally from each respondent prior to data collection in the local Turkana language, due to very low literacy rates. The data for Saba included here were derived from civil and parish birth, death, and marriage records, initially by P.W.L. in 1977 (historical records only; no research contact with living people), and updated by graduate students in 1984 and 2004, and supplemented by interviews with Sabian residents concerning migration. Informed consent was obtained from each respondent prior to interviews. Protocols for data collection and handling (including anonymization of data) were approved by the Institutional Review Boards of the State University of New York at Binghamton (1984) and the University of North Carolina at Chapel Hill (2004).

Sena; Mozambique (Mertens, Ziker) The data from central Mozambique included here were collected by Karl Mertens and John Ziker. Research permits were issued by Gorongosa National Park's Department of Scientific Services. Informed consent was obtained from each respondent prior to data collection. Following appropriate methods for non-literate populations, informed consent was obtained verbally after providing respondents with a verbal description (in Sena) of the research process and explaining how their data will be used (anonymously, for research purposes). Data collection protocols were approved by the Institutional Review Board at Boise State University (IRB #041-SB18-112).

Shodagor; Bangladesh (Starkweather) The fieldwork protocol was approved by the University of Missouri's Institutional Review Board, the Max Planck Institute for Evolutionary Anthropology's Department of Human Behaviour, Ecology and Culture, and the Ethical and Research Review Committees at the International Centre for Diarrhoeal Disease Research, Bangladesh (ICDDR,B). All respondents provided informed consent prior to completing interviews.

Shuar; Ecuador (Sugiyama, Madimenos, Liebert, Urlacher) The Shuar data were collected by L.S.S., F.M., M.L., and S.U., as part of a broader, longitudinal field study of the effects of market integration on Shuar health, life-history tradeoffs, and economy. The Federación Interprovincial de Centros Shuar authorized this research. Community leaders and members then authorized the research at open community meetings, during which a verbal description of the research was presented, and all questions answered and discussed (in Spanish, with Shuar translation and bilingual discussion). Due to high rates of non-literacy among older participants, as well as ongoing concerns over issues such as land rights, signing documents with non-Shuar

is potentially problematic for participants. Therefore, informed consent was obtained verbally from each participant prior to data collection. All procedures were approved by the University of Oregon Committee for the Protection of Human Subjects (CPHS), which serves as the UO Institutional Review Board (IRB).

Sukuma; Tanzania (Salerno) Sukuma data were collected by J.S. and four field assistants, I. Donald, V. Sintala, A. Sesa, and A. Thomas, from 2012 to 2013 in Katavi Region, Tanzania. Research ethics and protocols were approved by the Tanzanian Commission for Science and Technology (COSTECH: permit continuation 2017-251-NA-2017-16) and University of California Davis Institutional Review Board (IRB: 343343-1). Data collection was conducted in Kiswahili and Kisukuma. All participants gave verbal informed consent.

Tamil; India (Power) Data collection was undertaken by E.A.P. in 2013 and 2017 with help from the Chella Meenakshi Centre for Educational Research and Services and faculty and students at Madurai Kamaraj University. Fieldwork was approved by the Human Subjects Institutional Review Board of Stanford University and the University of Cincinnati. All participants provided verbal informed consent. The author is grateful for the patience and kindness of the villagers who participated in the study.

Tanna; Vanuatu (Atkinson) The Vanuatu data included here were collected by Q.D.A. with the permission and support of the Vanuatu Cultural Centre and the TAFEA Cultural Centre, and were approved by the University of Auckland Human Participants Ethics Committee (#9996).

Toba; Argentina (Valeggia) The Toba/Qom data included here were collected by C.V. and Dr. Norberto Lanza (deceased). The research protocol was approved by the Institutional Review Board of Harvard University (#F -11615-105) and by that of the University of Pennsylvania (Protocol #350000). The informed consent process was established with participants, all of whom spoke Spanish as a second language. All data were anonymized.

Tsimane' (Gurven et al.) Human subjects approval was granted by the institutional review boards at the University of New Mexico (HRRC #07–157; #15-133; #17-230) and University of California, Santa Barbara (HRRC #28-21-0788). Informed consent was established at three levels: individual, community, and Tsimane' government.

Twa; Angola (Davis) Demographic data collection methods were approved by the Institutional Review Board at Harvard University (IRB19-1401). Informed, verbal consent was established with communities and community members through the assistance of multilingual translators and guides.

Usangu (Sangu, Sukuma, Nyakyusa); Tanzania (McElreath) Field research in Usangu, Tanzania was approved by the Institutional Review Board at UCLA and by the Tanzanian Commission for Science and Technology (COSTECH). Informed consent was obtained verbally in Swahili. Consent was given only for anonymous data use.

Hadza, Lovu, Yasawa, Mauritius, Pesqueiro, Tanna, and Tuva; Tanzania, Fiji, Mauritius, Brazil, Vanuatu, and Siberia (Purzycki, Apicella, McNamara, Atkinson et al.) These data were collected under the auspices of the Evolution of Religion and Morality Project with ethics approval from the University of British Columbia's Behavioral Research Ethics Board (BREB #H13-00671). All participants gave informed verbal consent prior to participation, and were welcome to end participation at any time they wished.

The Hadza data collected by C.L.A. and local researchers were collected in 2013. All permits were approved by the Tanzanian Commission for Science and Technology (COSTECH Permit number 2013-55-ER-2000-80, entitled The Behavioural Ecology of Foraging in the Lake Eyasi Area). All procedures were approved by the University of Pennsylvania Institutional Review Board (protocol #818115). Consent was obtained verbally from each respondent prior to data collection in Kiswahili, due to limited literacy rates.

Efate, Tanna, Haiti, and Malawi; Vanuatu, Haiti, and Malawi (Mattison, Massengill, De-Marco, and Lanning) These data were collected as part of the Economic Networks and the Dynamics Of Wealth Inequality (ENDOW) project, with broad IRB approvals under the University of Cincinnati. The ENDOW project requires all contributing ethnographers to follow the American Anthropological Association's Statement on Ethics and its principles of professional responsibility without exception: do no harm, be open and honest regarding your work, obtain informed consent and necessary permissions, weigh competing ethical obligations due collaborators and affected parties, make your results accessible, protect and preserve your records, and maintain respectful and ethical professional relationships.

The data for Efate and Tanna were collected partially in association with the ENDOW project and partially supported by University of New Mexico under IRB approval 08116 and with additional review by the Vanuatu Cultural Centre. Voluntary informed consent was obtained verbally for all participants and a project information sheet was left for their reference.

For the Haiti study by A.L.D., data collection methods were approved by the Institutional Review Board at The University of Utah. Following local norms, the project was presented at a community meeting and approved by community leaders in May 2019. Subsequently, informed consent from individuals was obtained verbally prior to data collection.

The Malawian data included here were collected by J.L. and local research assistants as part of a wider field study on agricultural risk and decision-making. Data collection methods were approved by the Institutional Review Board at the University of Georgia (ID #CR00000256). Informed consent was obtained from each respondent prior to data collection, and from the community leader or family head, when appropriate. Due to limited literacy rates, informed consent was obtained verbally with the assistance of multilingual translators after providing respondents with a verbal description (in Chichewa) of the research process and explaining how their data will be used.

References

[1] James F Crow. Some possibilities for measuring selection intensities in man. *Human Biology*, 30(1):1–13, 1958.

[2] Michael J Wade and Stevan J Arnold. The intensity of sexual selection in relation to male sexual behaviour, female choice, and sperm precedence. *Animal Behaviour*, 28(2):446–461, 1980.

[3] Peter Nonacs. Measuring and using skew in the study of social behavior and evolution. *The American Naturalist*, 156(6):577–589, 2000.

[4] Peter Nonacs. Measuring the reliability of skew indices: Is there one best index? *Animal Behaviour*, 65(3):615–627, 2003.

[5] Joseph L Gastwirth. The estimation of the Lorenz curve and Gini index. *Review of Economics and Statistics*, pages 306–316, 1972.

[6] Nobuyuki Kutsukake and Charles L Nunn. Comparative tests of reproductive skew in male primates: The roles of demographic factors and incomplete control. *Behavioral Ecology and Sociobiology*, 60(5):695–706, 2006.

[7] Cody T Ross, Adrian V Jaeggi, Monique Borgerhoff Mulder, Jennifer E Smith, Eric Alden Smith, Sergey Gavrilets, and Paul L Hooper. The multinomial index: A robust measure of reproductive skew. *Proceedings of the Royal Society B*, 287(1936):20202025, 2020.

[8] Dieter Lukas and Tim Clutton-Brock. Costs of mating competition limit male lifetime breeding success in polygynous mammals. *Proceedings of the Royal Society B*, 281(1786): 20140418, 2014.

[9] Nathan S. Upham, Jacob A. Esselstyn, and Walter Jetz. Inferring the mammal tree: Species-level sets of phylogenies for questions in ecology, evolution, and conservation. *PLOS Biology*, 17(12):1–44, 12 2019.

[10] Mark Pagel. Inferring the historical patterns of biological evolution. *Nature*, 401(6756): 877–884, 1999.

[11] Simon P Blomberg, Theodore Garland Jr, and Anthony R Ives. Testing for phylogenetic signal in comparative data: Behavioral traits are more labile. *Evolution*, 57(4):717–745, 2003.

[12] Liam J Revell. Phytools: An R package for phylogenetic comparative biology (and other things). *Methods in Ecology and Evolution*, (2):217–223, 2012.

[13] P. David Polly. *Note: Phylogenetic comparative methods (PCM) for geometric morphometrics.* Earth and Atmospheric Sciences, Indiana University, 2018.

[14] Marguerite A Butler and Aaron A King. Phylogenetic comparative analysis: A modeling approach for adaptive evolution. *The American Naturalist*, 164(6):683–695, 2004.

[15] Cody T Ross, Pontus Strimling, Karen Paige Ericksen, Patrik Lindenfors, and Monique Borgerhoff Mulder. The origins and maintenance of female genital modification across Africa. *Human Nature*, 27(2):173–200, 2016.

[16] Riana Minocher, Pavel Duda, and Adrian V Jaeggi. Explaining marriage patterns in a globally representative sample through socio-ecology and population history: A Bayesian phylogenetic analysis using a new supertree. *Evolution and Human Behavior*, 40(2):176–187, 2019.

[17] Dieter Lukas, Mary Towner, and Monique Borgerhoff Mulder. The potential to infer the historical pattern of cultural macroevolution. *Philosophical Transactions of the Royal Society B*, 376(1828):20200057, 2021.

[18] Seung Yun Oh, Sam Bowles, and Monique Borgerhoff Mulder. The decline of polygyny: An interpretation. *Santa Fe Institute Working Paper*, 2017.

[19] Robert Dorfman. A formula for the Gini coefficient. *The Review of Economics and Statistics*, pages 146–149, 1979.

[20] Andreu Mas-Colell, Michael Dennis Whinston, Jerry R Green, et al. *Microeconomic theory*, volume 1. Oxford University Press, 1995.

[21] Jared Verner and Mary F Willson. The influence of habitats on mating systems of North American passerine birds. *Ecology*, 47(1):143–147, 1966.

[22] Gordon H Orians. On the evolution of mating system in birds and mammals. *American Naturalist*, pages 589–603, 1969.

[23] William A Searcy and Ken Yasukawa. Alternative models of territorial polygyny in birds. *The American Naturalist*, 134(3):323–343, 1989.

[24] Gaute Bø Grønstøl, Ingvar Byrkjedal, and Øyvind Fiksen. Predicting polygynous settlement while incorporating varying female competitive strength. *Behavioral Ecology*, 14(2):257–267, 2003.

[25] Eric D Gould, Omer Moav, and Avi Simhon. The mystery of monogamy. *The American Economic Review*, 98(1):333–357, 2008.

[26] George P Murdock and Douglas R White. Standard cross-cultural sample. *Ethnology*, 8 (4):329–369, 1969.

[27] J Patrick Gray. Ethnographic atlas codebook derived from George P. Murdock's ethnographic atlas recording the marital composition of 1231 societies from 1960 to 1980. *World Cultures*, 10(1):86–136, 1998.

[28] Mark V Flinn and Bobbi S Low. Resource distribution, social competition, and mating patterns in human societies. In D. I. Rubenstein and R. W. Wrangham, editors, *Ecological aspects of social evolution: Birds and mammals*, pages 217–243. Princeton University Press, 1986.

[29] Lucio Vinicius and Andrea Bamberg Migliano. Reproductive market values explain post-reproductive lifespans in men. *Trends in Ecology & Evolution*, 2016.

[30] Laura Betzig. Means, variances, and ranges in reproductive success: Comparative evidence. *Evolution and Human Behavior*, 33(4):309–317, 2012.

[31] Kyle Summers. The evolutionary ecology of despotism. *Evolution and Human Behavior*, 26(1):106–135, 2005.

[32] Tatiana Zerjal, Yali Xue, Giorgio Bertorelle, R Spencer Wells, Weidong Bao, Suling Zhu, Raheel Qamar, Qasim Ayub, Aisha Mohyuddin, Songbin Fu, et al. The genetic legacy of the Mongols. *The American Journal of Human Genetics*, 72(3):717–721, 2003.

[33] Beverly I Strassmann. Social monogamy in a human society: Marriage and reproductive success among the Dogon. In U. H. Reichard and C. Boesch, editors, *Monogamy: Mating Strategies and partnerships in Birds, Humans and Other Mammals*, pages 177–189. Cambridge University Press, 2003.

[34] Paul Spencer. Polygyny as a measure of social differentiation in Africa. In J.C. Mitchell, editor, *Numerical Techniques in Social Anthropology*. Philadelphia: Institute for the Study of Human Issues, 1980.

[35] Michael Cook. *A Brief History of the Human Race*. W. W. Norton, 2003.

[36] Monique Borgerhoff Mulder. Reproductive success in three Kipsigis cohorts. In T. H. Clutton-Brock, editor, *Reproductive Success: Studies of Individual Variation in Contrasting Breeding Systems*, pages 419–435. Princeton University Press, 1988.

[37] Alexandre Courtiol, Jenni E Pettay, Markus Jokela, Anna Rotkirch, and Virpi Lummaa. Natural and sexual selection in a monogamous historical human population. *Proceedings of the National Academy of Sciences*, 109(21):8044–8049, 2012.

[38] Kim Ronald Hill and A Magdalena Hurtado. *Ache Life History: The Ecology and Demography of a Foraging People*. Transaction Publishers, 1996.

[39] Woodrow W. Denham. *KinSources*. 2016. URL https://www.kinsources.net/.

[40] Cody T Ross, Patricia Joyas Campiño, and Bruce Winterhalder. Frequency-dependent social transmission and the interethnic transfer of female genital modification in the African diaspora and indigenous populations of Colombia. *Human Nature*, 26(4):351–377, 2015.

[41] Thomas Headland, Janet Headland, and Ray Uehara. *Agta Demographic Database: Chronicle of a hunter-gatherer community in transition*. 2011. URL http://www.sil.org/resources/publications/entry/9299.

[42] Abigail E Page, Emily H Emmott, Mark Dyble, Dan Smith, Nikhil Chaudhary, Sylvain Viguier, and Andrea B Migliano. Children are important too: Juvenile playgroups and maternal childcare in a foraging population, the Agta. *Philosophical Transactions of the Royal Society B*, 376(1827):20200026, 2021.

[43] Barry S Hewlett. *Intimate Fathers: The Nature and Context of Aka Pygmy Paternal Infant Care*. University of Michigan Press, 1993.

[44] Cristina Moya and Robert Boyd. The evolution and development of inferential reasoning about ethnic markers: Comparisons between urban United States and rural Highland Peru. *Current Anthropology*, 57(S13):S131–S144, 2016.

[45] Caroline Uggla, Eshetu Gurmu, and Mhairi A Gibson. Are wives and daughters disadvantaged in polygynous households? A case study of the Arsi Oromo of Ethiopia. *Evolution and Human Behavior*, 39(2):160–165, 2018.

[46] Stephen Beckerman and Roberto Lizarralde. *The Ecology of the Barí, Rainforest Horticulturalists of South America*. University of Texas Press, 2013.

[47] Adam H Boyette, Sheina Lew-Levy, Haneul Jang, and Vidrige Kandza. Social ties in the Congo Basin: insights into tropical forest adaptation from BaYaka and their neighbours. *Philosophical Transactions of the Royal Society B*, 377(1849):20200490, 2022.

[48] Mary K Shenk, Mary C Towner, Howard C Kress, and Nurul Alam. A model comparison approach shows stronger support for economic models of fertility decline. *Proceedings of the National Academy of Sciences*, 110(20):8045–8050, 2013.

[49] Mark A Caudell, Marsha B Quinlan, Murugan Subbiah, Douglas R Call, Casey J Roulette, Jennifer W Roulette, Adam Roth, Louise Matthews, and Robert J Quinlan. Antimicrobial use and veterinary care among agro-pastoralists in northern Tanzania. *PloS One*, 12(1): e0170328, 2017.

[50] Rebecca Sear. Kin and child survival in rural Malawi. *Human Nature*, 19(3):277–293, 2008.

[51] Shane J Macfarlan, Ryan Schacht, Eric Schniter, Juan José Garcia, Diego Guevara Beltran, and Jory Lerback. The role of dispersal and school attendance on reproductive dynamics in small, dispersed populations: Choyeros of Baja California Sur, Mexico. *PLOS One*, 15 (10):e0239523, 2020.

[52] Kathryn S Oths, Hannah N Smith, Max J Stein, and Rodrigo J Lazo Landivar. A decade of rapid change: Biocultural influences on child growth in highland Peru. *American Journal of Human Biology*, 30(2):e23072, 2018.

[53] John P Ziker. *Peoples of the Tundra: Northern Siberians in the Post-Communist Transition*. Waveland Press, 2002.

[54] Anne C Pisor, Matthew M Gervais, Benjamin G Purzycki, and Cody T Ross. Preferences and constraints: The value of economic games for studying human behaviour. *Royal Society Open Science*, 7(6):192090.

[55] Gregory Clark and Neil Cummins. Intergenerational wealth mobility in England, 1858–2012: Surnames and social mobility. *The Economic Journal*, 125(582):61–85, 2015.

[56] Virpi Lummaa, Jenni E Pettay, and Andrew F Russell. Male twins reduce fitness of female co-twins in humans. *Proceedings of the National Academy of Sciences*, 104(26): 10915–10920, 2007.

[57] Samuli Helle, Virpi Lummaa, and Jukka Jokela. Are reproductive and somatic senescence coupled in humans? Late, but not early, reproduction correlated with longevity in historical Sami women. *Proceedings of the Royal Society B*, 272(1558):29–37, 2005.

[58] Rebecca Sear, Nadine Allal, and Ruth Mace. Height, marriage and reproductive success in Gambian women. In *Socioeconomic aspects of human behavioral ecology*, pages 203–224. Emerald Group Publishing Limited, 2004.

[59] Frank Marlowe. *The Hadza: Hunter-Gatherers of Tanzania*. University of California Press, 2010.

[60] Brian M Wood and Frank Marlowe. Household and kin provisioning by Hadza men. *Human Nature*, 24(3):280–317, 2013.

[61] Nicholas Blurton Jones. *Demography and Evolutionary Ecology of Hadza Hunter-Gatherers*, volume 71. Cambridge University Press, 2016.

[62] Benjamin Grant Purzycki, Coren Apicella, Quentin D Atkinson, Emma Cohen, Rita Anne McNamara, Aiyana K Willard, Dimitris Xygalatas, Ara Norenzayan, and Joseph Henrich. Moralistic gods, supernatural punishment and the expansion of human sociality. *Nature*, 2016.

[63] Siobhán M. Mattison, Neil G. MacLaren, Ruizhe Liu, Adam Z. Reynolds, Gabrielle D. Baca, Peter M. Mattison, Meng Zhang, Chun-Yi Sum, Mary K. Shenk, Tami Blumenfield, Christopher von Rueden, and Katherine Wander. Gender differences in social networks based on prevailing kinship norms in the Mosuo of China. *Social Sciences*, 10(7), 2021.

[64] Brooke A Scelza. Female choice and extra-pair paternity in a traditional human population. *Biology Letters*, page 20110478, 2011.

[65] Kim Hill, A Magdalena Hurtado, and Robert S Walker. High adult mortality among Hiwi hunter-gatherers: Implications for human evolution. *Journal of Human Evolution*, 52(4): 443–454, 2007.

[66] Anne C Pisor and Michael Gurven. Risk buffering and resource access shape valuation of out-group strangers. *Scientific Reports*, 6:30435, 2016.

[67] Monique Borgerhoff Mulder. Reproductive success of three Kipsigis cohorts. In T. H. Clutton-Brock, editor, *Reproductive Success: Studies of Individual Variation in Contrasting Breeding Systems*, pages 419–435. University of Chicago Press, 1988.

[68] Awoke Amzaye Assoma. *Mosaic Cultures and Peoples of Southern Nations, Nationalities and Peoples' Regional State*. Awassa: Department of Culture, Bureau of Information and Culture, 2007.

[69] Kai Willführ and Charlotte Störmer. Social strata differentials in reproductive behavior among agricultural families in the Krummhörn region (East Frisia, 1720-1874). *Historical Life Course Studies*, 2:58–85, 2015.

[70] Nancy Howell. *Life Histories of the Dobe !Kung: Food, Fatness, and Well-being Over the Life Span*, volume 4. University of California Press, 2010.

[71] David A Nolin. Food-sharing networks in Lamalera, Indonesia: Status, sharing, and signaling. *Evolution and Human Behavior*, 33(4):334–345, 2012.

[72] Mark A Caudell, Marsha B Quinlan, Murugan Subbiah, Douglas R Call, Casey J Roulette, Jennifer W Roulette, Adam Roth, Louise Matthews, and Robert J Quinlan. Antimicrobial use and veterinary care among agro-pastoralists in Northern Tanzania. *PloS One*, 12(1): e0170328, 2017.

[73] Christopher D Golden, Cortni Borgerson, Benjamin L Rice, Lindsay H Allen, Evelin Jean Gasta Anjaranirina, Christopher B Barrett, Godfred Boateng, Jessica A Gephart, Daniela Hampel, Daniel L Hartl, et al. Cohort description of the Madagascar Health and Environmental Research–Antongil (MAHERY–Antongil) study in Madagascar. *Frontiers in Nutrition*, page 109, 2019.

[74] Ryan Schacht and Monique Borgerhoff Mulder. Sex ratio effects on reproductive strategies in humans. *Royal Society Open Science*, 2(1):140402, 2015.

[75] Tiffany E Mark, Ryan J Latulipe, Martina Anto-Ocrah, Geoffrey Mlongoti, David Adler, and Joseph W Lanning. Seasonality, food insecurity, and clinical depression in post-partum women in a rural Malawi setting. *Maternal and Child Health Journal*, 25(5):751–758, 2021.

[76] Caissa Revilla-Minaya. *Environmental factishes, variation, and emergent ontologies among the Matsigenka of the Peruvian Amazon.* Vanderbilt University, 2019.

[77] Luis Pacheco-Cobos and Bruce Winterhalder. Ethnographic observations on the role of domestic dogs in the lowland tropics of Belize with emphasis on crop protection and subsistence hunting. *Human Ecology*, 49(6):779–794, Dec 2021.

[78] Karen L Kramer and Garnett P McMillan. Women's labor, fertility, and the introduction of modern technology in a rural Maya village. *Journal of Anthropological Research*, 55(4): 499–520, 1999.

[79] Sean S Downey. Q'eqchi'Maya swidden agriculture, settlement history, and colonial enterprise in modern Belize. *Ethnohistory*, 62(4):751–779, 2015.

[80] Jeffrey Winking and Jeremy Koster. The fitness effects of men's family investments. *Human Nature*, 26(3):292–312, 2015.

[81] Eric Alden Smith, Rebecca Bliege Bird, and Douglas W Bird. The benefits of costly signaling: Meriam turtle hunters. *Behavioral Ecology*, 14(1):116–126, 2003.

[82] John Andrew Bunce and Richard McElreath. Interethnic interaction, strategic bargaining power, and the dynamics of cultural norms. *Human Nature*, pages 1–23, 2017.

[83] Anne C Pisor and Michael Gurven. When to diversify, and with whom? Choosing partners among out-group strangers in lowland Bolivia. *Evolution and Human Behavior*, 39(1): 30–39, 2018.

[84] Richard McElreath. Social learning and the maintenance of cultural variation: An evolutionary model and data from East Africa. *American Anthropologist*, 106(2):308–321, 2004.

[85] Monique Borgerhoff Mulder. Serial monogamy as polygyny or polyandry? Marriage in the Tanzanian Pimbwe. *Human Nature*, 20(2):130–150, 2009.

[86] Heidi Colleran and Ruth Mace. Social network-and community-level influences on contraceptive use: Evidence from rural Poland. *Proceedings of the Royal Society B: Biological Sciences*, 282(1807), 2015.

[87] Karen L Kramer and Russell D Greaves. Synchrony between growth and reproductive patterns in human females: Early investment in growth among Pumé foragers. *American Journal of Physical Anthropology*, 141(2):235–244, 2010.

[88] Julia A Jennings and Paul W Leslie. Differences in intergenerational fertility associations by sex and race in Saba, Dutch Caribbean, 1876–2004. *The History of the Family*, 18(2): 135–153, 2013.

[89] Samuli Helle, Jon E Brommer, Jenni E Pettay, Virpi Lummaa, Matti Enbuske, and Jukka Jokela. Evolutionary demography of agricultural expansion in preindustrial northern Finland. *Proceedings of the Royal Society B*, 281(1794):20141559, 2014.

[90] Kathrine E Starkweather. Shodagor family strategies. *Human Nature*, 28(2):138–166, 2017.

[91] Felicia C Madimenos, J Josh Snodgrass, Melissa A Liebert, Tara J Cepon, and Lawrence S Sugiyama. Reproductive effects on skeletal health in Shuar women of Amazonian Ecuador: A life history perspective. *American Journal of Human Biology*, 24(6):841–852, 2012.

[92] Robert J Quinlan, Samuel Jilo Dira, Mark Caudell, and M Quinlan. Culture and psychological responses to environmental shocks. *Current Anthropology*, 57(5), 2016.

[93] Craig Hadley, Monique Borgerhoff Mulder, and Emily Fitzherbert. Seasonal food insecurity and perceived social support in rural Tanzania. *Public Health Nutrition*, 10(06):544–551, 2007.

[94] Jonathan Salerno. Migrant decision-making in a frontier landscape. *Environmental Research Letters*, 11(4):044019, 2016.

[95] Eleanor A Power and Elspeth Ready. Cooperation beyond consanguinity: Post-marital residence, delineations of kin and social support among South Indian Tamils. *Philosophical Transactions of the Royal Society B*, 374(1780):20180070, 2019.

[96] Charles William Merton Hart, Arnold R Pilling, and Jane C Goodale. *The Tiwi of North Australia.* Holt, Rinehart and Winston, 1966.

[97] Norberto Lanza and Claudia Valeggia. Longitudinal analysis of reproductive behavior of a Toba transition population. *Revista Brasileira de Estudos de População*, 31(1):117–134, 2014.

[98] Michael Gurven, Hillard Kaplan, and Alfredo Zelada Supa. Mortality experience of Tsimane Amerindians of Bolivia: Regional variation and temporal trends. *American Journal of Human Biology*, 19(3):376–398, 2007.

[99] William R Leonard, Victoria Reyes-García, Susan Tanner, Asher Rosinger, Alan Schultz, Vincent Vadez, Rebecca Zhang, and Ricardo Godoy. The Tsimane' Amazonian Panel Study (TAPS): Nine years (2002–2010) of annual data available to the public. *Economics & Human Biology*, 19:51–61, 2015.

[100] Michael A Little and Paul W Leslie. *Turkana Herders of the Dry Savanna: Ecology and Biobehavioral Response of Nomads to an Uncertain Environment.* Oxford University Press Oxford:, 1999.

[101] Sevyan Vainshtein. *Nomads of South Siberia.* Cambridge University Press, 1980.

[102] James V Neel and Francisco M Salzano. Further studies on the Xavante Indians. *American Journal of Human Genetics*, 19(4):554, 1967.

[103] Cécile Vanpé. *Mating systems and sexual selection in ungulates. New insights from a territorial species with low sexual size dimorphism: The European roe deer (Capreolus capreolus).* PhD thesis, Université Paul Sabatier-Toulouse III; Swedish University of Agricultural Sciences, Uppsala, 2007.

[104] Loeske EB Kruuk, Tim H Clutton-Brock, Jon Slate, Josephine M Pemberton, Sue Brotherstone, and Fiona E Guinness. Heritability of fitness in a wild mammal population. *Proceedings of the National Academy of Sciences*, 97(2):698–703, 2000.

[105] J Slate, PM Visscher, S MacGregor, D Stevens, ML Tate, and JM Pemberton. A genome scan for quantitative trait loci in a wild population of red deer (*Cervus elaphus*). *Genetics*, 162(4):1863–1873, 2002.

[106] Anna Bess Sorin. Paternity assignment for white-tailed deer (*Odocoileus virginianus*): Mating across age classes and multiple paternity. *Journal of Mammalogy*, 85(2):356–362, 2004.

[107] Michael J Ford, KM Parsons, EJ Ward, JA Hempelmann, Candice K Emmons, M Bradley Hanson, Kenneth C Balcomb, and Linda K Park. Inbreeding in an endangered killer whale population. *Animal Conservation*, 21(5):423–432, 2018.

[108] TH Clutton-Brock, IR Stevenson, P Marrow, AD MacColl, AI Houston, and JM McNamara. Population fluctuations, reproductive costs and life-history tactics in female Soay sheep. *Journal of Animal Ecology*, pages 675–689, 1996.

[109] DW Coltman, JA Smith, DR Bancroft, J Pilkington, ADC MacColl, TH Clutton-Brock, and JM Pemberton. Density-dependent variation in lifetime breeding success and natural and sexual selection in Soay rams. *The American Naturalist*, 154(6):730–746, 1999.

[110] DW Coltman, M Festa-Bianchet, JT Jorgenson, and C Strobeck. Age-dependent sexual selection in bighorn rams. *Proceedings of the Royal Society of London B: Biological Sciences*, 269(1487):165–172, 2002.

[111] Jennifer D Cooper, Peter M Waser, Eric C Hellgren, Timothy M Gabor, and JA DeWoody. Is sexual monomorphism a predictor of polygynandry? Evidence from a social mammal, the collared peccary. *Behavioral Ecology and Sociobiology*, 65(4):775–785, 2011.

[112] Luca Corlatti, Bruno Bassano, Radka Poláková, Lorenzo Fattorini, Maria Chiara Pagliarella, and Sandro Lovari. Preliminary analysis of reproductive success in a large mammal with alternative mating tactics, the northern chamois, *Rupicapra rupicapra. Biological Journal of the Linnean Society*, 116(1):117–123, 2015.

[113] Michelle L Green, Denise L Herzing, and John D Baldwin. Reproductive success of male atlantic spotted dolphins (*Stenella frontalis*) revealed by noninvasive genetic analysis of paternity. *Canadian Journal of Zoology*, 89(3):239–253, 2011.

[114] Joanna Wiszniewski, Shannon Corrigan, Luciano B Beheregaray, and Luciana M Möller. Male reproductive success increases with alliance size in Indo-Pacific bottlenose dolphins (*Tursiops aduncus). Journal of Animal Ecology*, 81(2):423–431, 2012.

[115] E Natoli, M Schmid, L Say, and D Pontier. Male reproductive success in a social group of urban feral cats (*Felis catus L.*). *Ethology*, 113(3):283–289, 2007.

[116] Charlotte Henriette Marie Moueix et al. *Genetic verification of multiple paternity in two free-ranging isolated populations of African wild dogs (Lycaon pictus)*. PhD thesis, University of Pretoria, 2007.

[117] Ben T Hirsch and Jesus E Maldonado. Familiarity breeds progeny: Sociality increases reproductive success in adult male ring-tailed coatis (*Nasua nasua*). *Molecular Ecology*, 20 (2):409–419, 2011.

[118] Joseph I Hoffman, Ian L Boyd, and William Amos. Male reproductive strategy and the importance of maternal status in the Antarctic fur seal *Arctocephalus gazella. Evolution*, 57(8):1917–1930, 2003.

[119] Amanda M Sparkman, Jennifer Adams, Arthur Beyer, Todd D Steury, Lisette Waits, and Dennis L Murray. Helper effects on pup lifetime fitness in the cooperatively breeding red wolf (*Canis rufus*). *Proceedings of the Royal Society of London B: Biological Sciences*, 278 (1710):1381–1389, 2011.

[120] Kay E Holekamp, Jennifer E Smith, Christopher C Strelioff, Russell C Van Horn, and Heather E Watts. Society, demography and genetic structure in the spotted hyena. *Molecular Ecology*, 21(3):613–632, 2012.

[121] Hannah L Dugdale, David W Macdonald, Lisa C Pope, Paul J Johnson, and Terry Burke. Reproductive skew and relatedness in social groups of European badgers, *Meles meles*. *Molecular Ecology*, 17(7):1815–1827, 2008.

[122] Andrew M Robbins, Tara Stoinski, Katie Fawcett, and Martha M Robbins. Lifetime reproductive success of female mountain gorillas. *American Journal of Physical Anthropology*, 146(4):582–593, 2011.

[123] BJ Le Boeuf, Joanne Reiter, et al. Lifetime reproductive success in northern elephant seals. In T Clutton-Brock, editor, *Reproductive Success: Studies of Individual Variation in Contrasting Breeding Systems*, pages 344–362. University of Chicago Press, 1988.

[124] C Packer, L Herbst, AE Pusey, JD Bygott, JP Hanby, SJ Cairns, and M. Borgerhoff-Mulder. Reproductive success in lions. In T Clutton-Brock, editor, *Reproductive Success: Studies of Individual Variation in Contrasting Breeding Systems*, pages 363–383. University of Chicago Press,, 1988.

[125] DA Gilbert, Craig Packer, AE Pusey, J Claiborne Stephens, and Stephen J O'Brien. Analytical DNA fingerprinting in lions: Parentage, genetic diversity, and kinship. *Journal of Heredity*, 82(5):378–386, 1991.

[126] DW Coltman, WD Bowen, and Jonathan M Wright. Male mating success in an aquatically mating pinniped, the harbour seal (*Phoca vitulina*), assessed by microsatellite DNA markers. *Molecular Ecology*, 7(5):627–638, 1998.

[127] Sean A Hayes, Devon E Pearse, Daniel P Costa, James T Harvey, Burney J Le Boeuf, and John Carlos Garza. Mating system and reproductive success in eastern Pacific harbour seals. *Molecular Ecology*, 15(10):3023–3034, 2006.

[128] Göran F Spong, Sarah J Hodge, Andrew J Young, and Tim H Clutton-Brock. Factors affecting the reproductive success of dominant male meerkats. *Molecular Ecology*, 17(9): 2287–2299, 2008.

[129] Adrienne I Kovach and Roger A Powell. Effects of body size on male mating tactics and paternity in black bears, *Ursus americanus*. *Canadian Journal of Zoology*, 81(7):1257–1268, 2003.

[130] Ulrich Pörschmann, Fritz Trillmich, Birte Mueller, and Jochen BW Wolf. Male reproductive success and its behavioural correlates in a polygynous mammal, the Galapagos sea lion (*Zalophus wollebaeki). Molecular Ecology*, 19(12):2574–2586, 2010.

[131] Jay F Storz, Hari R Bhat, and Thomas H Kunz. Genetic consequences of polygyny and social structure in an Indian fruit bat, *Cynopterus sphinx*. Inbreeding, outbreeding, and population subdivision. *Evolution*, 55(6):1215–1223, 2001.

[132] Gerald Kerth, Kamran Safi, and Barbara König. Mean colony relatedness is a poor predictor of colony structure and female philopatry in the communally breeding Bechstein's bat (*Myotis bechsteinii*). *Behavioral Ecology and Sociobiology*, 52(3):203–210, 2002.

[133] FJL Kraaijeveld-Smit, SJ Ward, and PD Temple-Smith. Paternity success and the direction of sexual selection in a field population of a semelparous marsupial, *Antechinus agilis*. *Molecular Ecology*, 12(2):475–484, 2003.

[134] Clare E Holleley, CR Dickman, Mathew S Crowther, and BP Oldroyd. Size breeds success: Multiple paternity, multivariate selection and male semelparity in a small marsupial, *Antechinus stuartii*. *Molecular Ecology*, 15(11):3439–3448, 2006.

[135] SL Hazlitt, DP Sigg, MDB Eldridge, and AW Goldizen. Restricted mating dispersal and strong breeding group structure in a mid-sized marsupial mammal (*Petrogale penicillata*). *Molecular Ecology*, 15(10):2997–3007, 2006.

[136] William AH Ellis and Fred B Bercovitch. Body size and sexual selection in the koala. *Behavioral Ecology and Sociobiology*, 65(6):1229–1235, 2011.

[137] Cole Burton, Charles J Krebs, and Eric B Taylor. Population genetic structure of the cyclic snowshoe hare (*Lepus americanus*) in southwestern Yukon, Canada. *Molecular Ecology*, 11(9):1689–1701, 2002.

[138] Petra Kretzschmar, Hailie Auld, Peter Boag, Udo Gansloßer, Candace Scott, Peter John Van Coeverden de Groot, and Alexandre Courtiol. Mate choice, reproductive success and inbreeding in white rhinoceros: New insights for conservation management. *Evolutionary applications*, 13(4):699–714, 2020.

[139] Julie N Garnier, Michael William Bruford, and Benoit Goossens. Mating system and reproductive skew in the black rhinoceros. *Molecular Ecology*, 10(8):2031–2041, 2001.

[140] Daniel I Rubenstein and Cassandra M Nuñez. Sociality and reproductive skew in horses and zebras. In Reinmar Hager and Clara B Jones, editors, *Reproductive Skew in Vertebrates: Proximate and Ultimate Causes*, pages 196–226. Cambridge University Press, 2009.

[141] Jonathan N Pauli and M Zachariah Peery. Unexpected strong polygyny in the brown-throated three-toed sloth. *PLoS One*, 7(12):e51389, 2012.

[142] Karen B Strier, Jean P Boubli, Carla B Possamai, and Sérgio L Mendes. Population demography of northern muriquis (*Brachyteles hypoxanthus*) at the Estação Biológica de Caratinga/Reserva Particular do Patrimônio Natural-Feliciano Miguel Abdala, Minas Gerais, Brazil. *American Journal of Physical Anthropology*, 130(2):227–237, 2006.

[143] Susan Perry, Irene Godoy, and Wiebke Lammers. The Lomas Barbudal Monkey Project: Two decades of research on *Cebus capucinus*. In *Long-term Field Studies of Primates*, pages 141–163. Springer, 2012.

[144] DL Cheney, RM Seyfarth, SJ Andelman, and PC Lee. Reproductive success in vervet monkeys. In TH Clutton-Brock, editor, *Reproductive Success: Studies of Individual Variation in Contrasting Breeding Systems*, pages 384–402. University of Chicago Press, 1988.

[145] Claudia Barelli, Kazunari Matsudaira, Tanja Wolf, Christian Roos, Michael Heistermann, Keith Hodges, Takafumi Ishida, Suchinda Malaivijitnond, and Ulrich H Reichard. Extra-pair paternity confirmed in wild white-handed gibbons. *American Journal of Primatology*, 75(12):1185–1195, 2013.

[146] MaLinda D Henry, Sarah J Hankerson, Jennifer M Siani, Jeffrey A French, and James M Dietz. High rates of pregnancy loss by subordinates leads to high reproductive skew in wild golden lion tamarins (*Leontopithecus rosalia*). *Hormones and Behavior*, 63(5):675–683, 2013.

[147] Miho Inoue, Akiko Takenaka, Shoji Tanaka, Ryo Kominami, and Osamu Takenaka. Paternity discrimination in a Japanese macaque group by DNA fingerprinting. *Primates*, 31(4): 563–570, 1990.

[148] Ulrike Sauermann, Peter Nürnberg, Fred Bercovitch, John Berard, Andrea Trefilov, Anja Widdig, Matt Kessler, Jörg Schmidtke, and Michael Krawczak. Increased reproductive success of MHC class II heterozygous males among free-ranging rhesus macaques. *Human Genetics*, 108(3):249–254, 2001.

[149] Constance Dubuc, Laura Muniz, Michael Heistermann, Antje Engelhardt, and Anja Widdig. Testing the priority-of-access model in a seasonally breeding primate species. *Behavioral Ecology and Sociobiology*, 65(8):1615–1627, 2011.

[150] James P Higham, Michael Heistermann, Muhammad Agil, Dyah Perwitasari-Farajallah, Anja Widdig, and Antje Engelhardt. Female fertile phase synchrony, and male mating and reproductive skew, in the crested macaque. *Scientific Reports*, 11(1):1–11, 2021.

[151] Jutta Kuester, Andreas Paul, and Joachim Arnemann. Age-related and individual differences of reproductive success in male and female Barbary macaques (*Macaca sylvanus*). *Primates*, 36(4):461–476, 1995.

[152] Ulrike Gerloff, Bianka Hartung, Barbara Fruth, Gottfried Hohmann, and Diethard Tautz. Intracommunity relationships, dispersal pattern and paternity success in a wild living community of bonobos (*Pan paniscus*) determined from DNA analysis of faecal samples. *Proceedings of the Royal Society B*, 266(1424):1189–1195, 1999.

[153] Grit Schubert, Linda Vigilant, Christophe Boesch, Reinhard Klenke, Kevin Langergraber, Roger Mundry, Martin Surbeck, and Gottfried Hohmann. Co–residence between males and their mothers and grandmothers is more frequent in bonobos than chimpanzees. *PloS One*, 8(12):e83870, 2013.

[154] Martin Surbeck, Kevin E Langergraber, Barbara Fruth, Linda Vigilant, and Gottfried Hohmann. Male reproductive skew is higher in bonobos than chimpanzees. *Current Biology*, 27:R623–R641, 2017.

[155] Pascal Gagneux, Christophe Boesch, and David S Woodruff. Female reproductive strategies, paternity and community structure in wild West African chimpanzees. *Animal Behaviour*, 57(1):19–32, 1999.

[156] Eiji Inoue, Miho Inoue-Murayama, Linda Vigilant, Osamu Takenaka, and Toshisada Nishida. Relatedness in wild chimpanzees: Influence of paternity, male philopatry, and demographic factors. *American Journal of Physical Anthropology*, 137(3):256–262, 2008.

[157] Nicholas E Newton-Fisher, Melissa Emery Thompson, Vernon Reynolds, Christophe Boesch, and Linda Vigilant. Paternity and social rank in wild chimpanzees (*Pan troglodytes*) from the Budongo Forest, Uganda. *American Journal of Physical Anthropology*, 142(3):417–428, 2010.

[158] Emily E Wroblewski, Carson M Murray, Brandon F Keele, Joann C Schumacher-Stankey, Beatrice H Hahn, and Anne E Pusey. Male dominance rank and reproductive success in chimpanzees, *Pan troglodytes schweinfurthii*. *Animal Behaviour*, 77(4):873–885, 2009.

[159] Susan C Alberts, Jason C Buchan, and Jeanne Altmann. Sexual selection in wild baboons: From mating opportunities to paternity success. *Animal Behaviour*, 72(5):1177–1196, 2006.

[160] Richard R Lawler. Fitness and extra-group reproduction in male Verreaux's sifaka: An analysis of reproductive success from 1989–1999. *American Journal of Physical Anthropology*, 132(2):267–277, 2007.

[161] Toni Lyn Morelli. *Dispersal, kinship, and genetic structure of an endangered Madagascar primate, Propithecus edwardsi.* State University of New York at Stony Brook, 2008.

[162] Henrik Barner Rasmussen, JBA Okello, G Wittemyer, Hans Redlef Siegismund, Peter Arctander, F Vollrath, and Ian Douglas-Hamilton. Age-and tactic-related paternity success in male African elephants. *Behavioral Ecology*, 19(1):9–15, 2007.

[163] Priyank Sharad Nimje, Helga Veronica Tinnesand, Christina Buesching, Mona Sæbø, Helen Senn, Andreas Zedrosser, and Frank Rosell. Almost faithful: SNP markers reveal low levels of extra-pair paternity in the Eurasian beavers. *PeerJ Preprints*, 7:e27866v1, 2019.

[164] Albrecht I Schulte-Hostedde, John S Millar, and H Lisle Gibbs. Sexual selection and mating patterns in a mammal with female-biased sexual size dimorphism. *Behavioral Ecology*, 15(2):351–356, 2004.

[165] Patrick Bergeron, Pierre-Olivier Montiglio, Denis Réale, Murray M Humphries, and Dany Garant. Bateman gradients in a promiscuous mating system. *Behavioral Ecology and Sociobiology*, 66(8):1125–1130, 2012.

[166] Tamsin M Burland, Nigel C Bennett, Jennifer UM Jarvis, and Christopher G Faulkes. Colony structure and parentage in wild colonies of co-operatively breeding damaraland mole-rats suggest incest avoidance alone may not maintain reproductive skew. *Molecular Ecology*, 13(8):2371–2379, 2004.

[167] David O Ribble. Lifetime reproductive success and its correlates in the monogamous rodent, *Peromyscus californicus*. *Journal of Animal Ecology*, pages 457–468, 1992.

[168] Matthew D. Hoffman and Andrew Gelman. The No-U-Turn Sampler: Adaptively setting path lengths in Hamiltonian Monte Carlo. *Journal of Machine Learning Research*, 2014.

[169] Stan Development Team. Stan: A C++ library for probability and sampling, version 2.16.0, 2017. URL http://mc-stan.org/.

[170] R Core Team. *R: A Language and Environment for Statistical Computing*. R Foundation for Statistical Computing, Vienna, Austria, 2015. URL https://www.R-project.org/.
